# Evolution of genomic architecture of the plant-pathogenic fungus Alternaria revealed by comparative analyses of 12 chromosome-level assemblies

**DOI:** 10.1099/mgen.0.001686

**Published:** 2026-04-10

**Authors:** Jeremy R. Dettman, Natalie E. Kim, Kasia Dadej

**Affiliations:** 1 Agriculture and Agri-Food Canada, Ottawa Research and Development Centre, Ottawa, ON, Canada

**Keywords:** *Alternaria alternata*, *Alternaria arborescens*, genome compartmentalization, subtelomeres

## Abstract

Gapless, chromosome-level assemblies provide unparalleled resolution for studying the architecture and evolution of genomes. We produced new telomere-to-telomere genome assemblies for five strains representing four species from the plant-pathogenic fungal genus *Alternaria*, focusing on section *Alternaria*. These new genomic resources were combined with seven previously published assemblies, allowing for detailed comparative analyses of genome architecture, chromosome structure and associated evolutionary dynamics. All strains possessed a stable complement of ten core chromosomes with highly conserved macrosynteny; only a few large-scale structural rearrangements were observed. Consensus genomic features, such as chromosome length, centromeres, subtelomeres, GC composition, gene density and repetitive content, were characterized in depth and were highly similar in most genomes. A metric for quantifying inter-genomic orthogroup retention revealed a consistent gradient of elevated homologue gain/loss toward chromosome ends. The increased rate of gene turnover is an inherent property of these dynamic regions and is not driven by the enrichment of certain functional classes of embedded genes. Despite the plasticity of chromosome ends, multiple complementary analyses found no evidence for physical or functional bipartite (or ‘two-speed’) compartmentalization as a whole. For example, candidate effector genes did not display biased localization to gene-sparse regions. Collectively, our results demonstrate that *Alternaria* section *Alternaria* has a conserved, stable genomic architecture yet retains evolutionarily dynamic regions localized to chromosomal termini. These gapless genomes provide a framework to support further studies on chromosomal evolution and pathogenicity-related diversification in this economically important fungal group.

**Impact Statement**

Fungal species in *Alternaria* section *Alternaria* commonly cause disease on a wide range of agricultural crops, yet little is known about how their genomes are organized. The evolution of certain genomic characteristics may help a species be a successful pathogen, but this has not been studied in section *Alternaria*. Here, we perform the first comprehensive analysis of multiple, high-quality genomes from this economically important group of fungi. We find that the large-scale genomic architecture is very similar across all analysed strains, but the ends of chromosomes are evolutionarily dynamic regions that experience elevated rates of gene gain or loss. Other genomic patterns often associated with plant pathogens were not detected. Our results demonstrate how the evolution of genomic architecture of fungal pathogens may be diverse and complex, suggesting that broader taxonomic sampling is needed before generalized conclusions can be made with confidence.

## Data Summary

The genome assemblies that were newly generated for the current study, and the other assemblies derived from sources in the public domain, are available from the National Center for Biotechnology Information (NCBI; https://www.ncbi.nlm.nih.gov) under the accession numbers listed in Table 1. Raw long-read sequence data are available from the NCBI Sequence Read Archive under BioProject PRJNA702449.

## Introduction

The accurate sequence of a complete genome, along with a comprehensive catalogue of genomic features, has become a fundamental resource for research on the evolution and genetics of a specific organism. Advancements in high-throughput sequencing technologies have led to a rapid increase in the number of whole-genome assemblies available for most taxonomic groups, including the fungal kingdom [1]. The vast majority are draft assemblies generated from short-read sequence data, with the genome being fragmented into hundreds or thousands of contigs. Repetitive regions or identical duplications that are longer than the sequence read length are particularly challenging for most short-read assembly algorithms, and the failure to recover such genomic regions in the final assembly results in assembly gaps [2,3]. While these fragmented assemblies are often sufficient for capturing most of the genome’s unique gene content, they typically cannot be used for accurately resolving features such as chromosome lengths, centromere locations and repetitive element catalogues. Thus, draft assemblies are usually not adequate for detailed inference of genomic architecture or structural evolution of chromosomes [4].

With continued developments in long-read (e.g. Oxford Nanopore and PacBio) and long-range (e.g. Hi-C) sequencing technologies, researchers are now able to generate gapless, complete, chromosome-level assemblies for most types of organisms. Long-read sequences can span the repetitive or duplicated regions that are difficult for short-read data to resolve, allowing assembly of full-length chromosomes and truly complete genomes [3]. These chromosome-level assemblies can extend from telomere to telomere, allowing researchers to determine the exact chromosomal locations and orientations of all genomic features of interest. The structural accuracy, repeat resolution and comprehensive content annotation of gapless genomes make them the benchmark for comparative and functional genomic research.

Gapless genomes are being generated for a wide range of fungal species [e.g. 5,7], providing high-quality genomic data to support further research. While single-genome resources are undoubtedly useful, multiple genomes obtained from several individuals are needed to address some of the broader questions in fungal genome evolution: Is the genomic architecture found in one individual also conserved across all individuals in the species, or closely related species? How do genomic architecture and chromosomal structure evolve over short or long timescales? A common approach is to establish a complete ‘reference’ genome (preferably gapless), then map additional draft genomes or sequence data back to the reference to identify sequence or structural variation. In these cases, the position and orientation of draft contigs are inferred by assumed homology to the reference chromosomes. One problem is that chromosomal rearrangement breakpoints often coincide with repetitive genomic regions represented by assembly gaps [2,8], compounding the difficulty in accurately determining the placement and orientation of draft assembly contigs. While the default assumption for positional and directional synteny is reasonable, it would not hold true for any species that experiences extensive chromosomal rearrangements or reshuffling [9]. Therefore, comparative analysis of multiple gapless genomes is required to confidently characterize the evolution of chromosomal architecture.

Recent studies that have included multiple gapless genomes from the same or closely related fungal species have reported drastically different patterns of variation in genomic architecture. Regarding the conservation of chromosomal structure within species, for example, some species displayed fairly high levels of chromosomal macrosynteny across strains, with only rare cases of chromosomal rearrangements [10,12]. In contrast, strains from other species can have frequent major chromosomal rearrangements, including inter-chromosomal translocations [9,13, 14]. Significantly different results have also been obtained regarding evidence for bipartite genome compartmentalization, or the ‘two-speed’ model of genome evolution [15,17]. This model posits that the genomes of fungal pathogens may contain distinct repeat-rich, gene-poor regions that experience higher rates of evolution due, in part, to reduced purifying selection and increased mutation rates. These regions are considered evolutionary hotspots that drive adaptation via increased diversification of effectors and other pathogenicity- or virulence-related genes that are often located within such regions. While this model was built upon clear examples of strong bipartite structure in some fungal species [18,19], many similar investigations of other fungal pathogens have failed to find evidence of compartmentalization [20,24]. A broader sampling of fungal taxa is needed before general predictions can be made based on taxonomy or lifestyle.

Species in the large, cosmopolitan genus *Alternaria* are associated with a broad diversity of niches and a wide range of lifestyles [25,26]. Some species may function as saprobes that decay organic matter, while others may form harmless endo/epiphytic relationships with host plants. More importantly, many species are economically detrimental plant pathogens that cause disease on hundreds of globally produced agricultural crops [27,28]. Several of these notorious plant-pathogenic species are classified within the sub-generic *Alternaria* section *Alternaria* [25,26, 29], including *Alternaria alternata*, one of the most commonly encountered of all *Alternaria* species.

In this study, we performed comparative analyses of gapless, chromosome-level genomes of 12 strains from four species in *Alternaria* section *Alternaria*, with the goal of establishing a solid framework for further targeted investigations on genome organization and evolution in this plant-pathogenic fungal genus. We also explored concepts that could only be addressed using gapless genomes. Some gapless assemblies were previously produced for strains within section *Alternaria* [30,34]; however, all of them were from one species, *A. alternata*. We generated new gapless genomes for two additional *A. alternata* strains, and the first gapless genomes for three other closely related species: *Alternaria arborescens*, *Alternaria longipes* and a recently discovered, undescribed *Alternaria* species. Patterns of genomic macrosynteny were assessed to determine if chromosomal structure was conserved within and/or across species. Analyses of genome-wide sequence similarity and shared orthogroups revealed the phylogenetic relationships among strains. Key physical features of the genomes were characterized to establish the consensus genomic architecture for this group, and the association of different genomic features was examined at the chromosomal level. We calculated a metric called ‘inter-genomic orthogroup retention’ (IGOR) to study the gene-by-gene patterns of homologue gain/loss across genomes and along chromosomes. Finally, we searched for evidence of bipartite compartmentalization in representative genomes of each species.

## Methods

### Strains and DNA extraction

The live fungal strains included in this study are part of the Agriculture and Agri-Food Canada mycological research collections held in Ottawa, Ontario, Canada. Prefixes of KAS or DET indicate that the source was the Keith Seifert or Dettman laboratory, respectively. Strains were accessioned for long-term storage in the Canadian Collection of Fungal Cultures (DAOMC), with the corresponding DAOMC numbers listed in Table 1. Cultures were grown on potato dextrose agar (potato extract, 4 g l^−1^; dextrose, 20 g l^−1^; agar, 15 g l^−1^) and incubated for 10–14 days at 25 ˚C. Genomic DNA was extracted from homogenized tissue using a cetyltrimethylammonium bromide buffer for cell lysis, chloroform:isoamyl alcohol for organic purification, isopropanol for precipitation and ethanol for washing. The detailed protocol for DNA extraction was previously described in [35].

**Table 1.** Information on fungal strains and genome assemblies. New assemblies are indicated in bold

Strain	Species	Isolation source (host, country, year)	Genome accession	Reference	BUSCO completeness (%)	Assembly size (Mb) of all 10 core chromosomes	GC %	Total predicted gene models
DZ	*A. alternata*	*Nicotiana tabacum*, China, 2020	GCA_029891365.1	[34]	99.1	34.11	50.95	11,840
JS-0527	*A. alternata*	*Phragmites australis*, South Korea, 2014	GCA_011420255.1 (WIRD01)	na	99.0	33.50	50.98	11,793
**KAS5386 (DAOMC252607)**	***A. alternata***	***Calamagrostis*, Canada, 2014**	**JAFFSA000000000**	**This study**	**99.1**	**33.49**	**51.00**	**11,696**
**KAS5497 (DAOMC252608)**	***A. alternata***	***Brassica napus*, Canada, 2013**	**JBGLPV000000000**	**This study**	**99.0**	**33.95**	**51.07**	**11,839**
NAP07	*A. alternata*	*Prunus persica*, Japan	GCA_009932595.1 (BJEP01)	[31]	98.9	33.81	51.02	11,724
PN1	*A. alternata*	*Brassica juncea*, India, 2017	GCA_011420445.1 (VEOJ01)	[30]	98.9	33.63	51.02	11,766
PN2	*A. alternata*	*Brassica juncea*, India, 2017	GCA_011420565.1 (VEOK01)	[30]	98.9	33.25	51.03	11,756
Y784-BC03	*A. alternata*	*Actinidia chinensis*, China, 2020	GCA_020085065.1 (JAHEWI01)	[33]	99.0	33.77	50.99	11,761
Z7	*A. alternata*	*Citrus reticulata*, China	GCA_014751505.1	[32]	98.9	33.30	51.03	11,643
**KAS1274**	***A. longipes***	***Astronium suaveolens*, Costa Rica, 1999**	**JBGLPU000000000**	**This study**	**98.9**	**33.75**	**50.94**	**11,732**
**KAS6096 (DAOMC251703)**	**Undescribed species**	**Indoor dust, Canada, 2015**	**JBGLPW000000000**	**This study**	**99.1**	**35.86**	**50.10**	**11,866**
**DET2035 (DAOMC252606)**	***A. arborescens***	***Triticum*, Canada, 2018**	**JAFFSB000000000**	**This study**	**98.8**	**33.17**	**51.09**	**11,567**

### Sequence data generation

For long-read sequencing, whole-genome libraries were prepared from high-molecular-weight genomic DNA using the Ligation Sequencing kit SQK-LSK109 (Oxford Nanopore Technologies, UK). Libraries were sequenced on a FLO-MIN106 (R9.4.1) flow cell using a MinION device (Oxford Nanopore Technologies, UK) for a duration of 48 h, following the manufacturer’s protocols. Conversion from fast5 to fastq format (basecalling) was performed with GUPPY (4.2.2; https://nanoporetech.com/community) under the high accuracy (hac) model and other settings on default. For short-read sequencing, genomic DNA was normalized to 300 ng and sheared to a ~350 bp fragment size using a Covaris M220 focused-ultrasonicator (Covaris, Massachusetts). The resulting DNA was used as the template to construct PCR-free libraries with a NxSeq AmpFREE Low DNA Library Kit (Lucigen, Wisconsin). Indexed libraries were sequenced with paired-end reads (2×150 bp) on a NextSeq500 instrument (Illumina, California) using a NextSeq High Output Reagent Kit v2.5 (300 cycles; Illumina), according to the manufacturer’s instructions. The sequencing work was performed at the Molecular Technologies Laboratory at Ottawa Research and Development Centre, Agriculture and Agri-Food Canada. Sequence data outputs are summarized in Table S1, available in the online Supplementary Material.

### Genome assembly

Nanopore reads were corrected, *de novo* assembled and bridged into contigs using NECAT (0.0.1 [36]). All settings were default, except MIN_READ_LENGTH was 1000 and GENOME_SIZE was set to the estimated size of the genome. Three different combinations of PREP_OUTPUT_COVERAGE and CNS_OUTPUT_COVERAGE were tested for each genome to accommodate potential differences in total data input. The most contiguous of the three resulting assembly versions was retained for subsequent polishing (Table S2). Four successive rounds of polishing with Nanopore reads were performed with Racon (1.5.0 [37]), and an additional four successive rounds of polishing with Illumina reads were performed with Pilon (1.23 [38]; Text S1). Genome assembly from long-read data was also performed with CANU (2.1.1 [39]) with genomeSize=34m, correctedErrorRate=0.154 and minOverlapLength=1000, and all other settings set to default. CANU assemblies were used to cross-validate NECAT assemblies (Table S3). Assembly completeness was assessed with BUSCO (5.7.0 [40]) based on the genome sequence <-m genome> and the ascomycota_odb10 database.

### Existing genome data

Seven chromosome-level assemblies for *A. alternata* strains were downloaded from the NCBI public repository (Table 1; accessed 4 April 2024). To represent the outgroup, the genome and protein sequences for *Alternaria solani* strain BMP0185 were downloaded from the JGI MycoCosm portal (https://mycocosm.jgi.doe.gov/Altso1/Altso1.home.html).

### Macrosynteny and sequence similarity of shared genomic regions

Comparison of chromosome structure and macrosynteny was performed with the NUCMER module of MUMMER (4.0 [41]). Maximal unique matches (MUMs) were determined using a minimum cluster size of 1,000 bases <-c 1000> for all possible, reciprocal pairwise comparisons between strains. The coordinates of matching regions between genomes were outputted using the show-coords function <-r -c -l -d>, and dotplots of MUMs were generated with the mummerplot function. To avoid redundancy, only one of the two reciprocal analyses of each genome pair is reported. NUCMER results were parsed to calculate the overall average sequence similarity of all genomic regions shared between two strains. This value is a useful comparative metric but may slightly overestimate the total genomic sequence similarity because it does not include the small proportion of genomic regions that are unique to one strain.

### Feature prediction and annotation

Gene prediction and annotation were performed using FUNANNOTATE (1.8.15; https://github.com/nextgenusfs/funannotate) following the methods described in [35]. In brief, training was performed on the *A. alternata* strain SRC1lrk2f with associated RNASeq transcriptomic data as additional evidence. Gene prediction was performed with Augustus, Genemark, PASA, SNAP and GlimmerHMM, using pre-trained parameters when appropriate, and the resulting gene models were reconciled with EvidenceModeler. Final gene predictions were analysed with antiSMASH (7.1.0 [42]) to identify secondary metabolite biosynthetic gene clusters (BGCs). BGC counts included those in the polyketide synthase (PKS), non-ribosomal peptide synthetase (NRPS), PKS-NRPS hybrid, terpene and ribosomally synthesized and post-translationally modified peptide classes. Putative centromeres were identified as large isolated regions of AT-rich, transposon-rich sequence that were located in similar locations across homologous chromosomes. Telomeres were identified by searching with canonical telomeric repeat sequences (TTAGGG/CCCTAA). AT-rich tracts were identified using OcculterCut (1.1 [43]) with default settings. Data for GC-content plots were extracted from OcculterCut outputs. Regions of the genome affected by repeat-induced point (RIP) mutation were predicted by RIPper (1.0 [44]) with a window and step size of 1,000 and 500 bp, respectively. Transposable and repetitive elements (TREs) were identified and annotated using RepeatModeler (2.0.1 [45]) and RepeatMasker (4.1.2-p1). A RepeatModeler BLAST database was built for each genome with the <BuildDatabase> command, then <RepeatModeler> was run on the new database to *de novo* identify repetitive elements. The library was split into successfully classified elements and those that remained as unknown elements. Repeat annotation and masking were performed with RepeatMasker in four consecutive rounds, with results from one round being used as the input for the next round. Repeat types were annotated in priority of prediction robustness: (i) low complexity/simple repeats, (ii) well-known complex repeats from the RepBase library (20181026 Edition), (iii) known complex repeats identified by RepeatModeler and then (iv) unknown repeats identified by RepeatModeler (Text S2). Annotation results from the four rounds were concatenated and processed with the <ProcessRepeats> utility. The <-species fungi> and <-a> flags were called to indicate taxon and save alignment information, respectively. To identify candidate effectors, all proteins were first analysed with SignalP (6 [46]) to detect eukaryotic <-- organism euk> signal peptides (Sec/SPI). Proteins with signal peptides were then analysed with EffectorP (3.0.0-beta [47]) to identify putative effectors. Finally, the putative effector proteins were analysed with DeepTMHMM (1.0.44 [48]), and those with predicted transmembrane domains were excluded.

### Phylogenomic analyses

Inference of orthogroups based on all predicted proteins was performed with OrthoFinder (2.5.5 [49]) under default settings. Inference of phylogenetic relationships among taxa included the 12 ingroup strains and 1 outgroup, *A. solani* strain BMP0185. This species was chosen because it is classified within *Alternaria* section *Porri*, the most closely related section to the ingroup, section *Alternaria*. OrthoFinder analyses were run on protein sequence files to identify core single-copy orthogroups, which were then aligned with MUSCLE [50]. Model selection and construction of maximum likelihood phylogenies were performed with IQ-TREE (2.0 [51]). Tree construction was performed under the best-fit substitution models and the optimal partitioning scheme <-m MFP+MERGE>, as determined by the ModelFinder module. Branch support was assessed using 1,000 replicates of ultrafast bootstrapping <-ufboot 1000>.

### Detailed analyses of features on homologous chromosomes

OrthoFinder orthogroup results were parsed to calculate the ‘inter-genomic orthogroup retention’ (IGOR) value for each gene in each genome (Text S3). The IGOR value equals the proportion of other analysed genomes that possessed an identified homologue of the focal gene, regardless of the chromosomal location of the homologues. The midpoint coordinates of genes and TREs were used when comparing the locations of these features along the lengths of homologous chromosomes. For BGC location calculations, the midpoint coordinate of the entire BGC was used. Relative locations ranged from 0 to 100, from the left to the right terminus of each chromosome.

Calculation of distances between features used their 3′ and 5′ boundaries. For gene and effector density compartmentalization, annotated gff3 feature files were passed to the genome_speed_hexbins.py script [52] which creates a hexbin plot of 5′ and 3′ intergenic distances. The first and last genes from each chromosome were excluded because they are missing data for one axis. Effectors were overlain onto the distribution of all other genes, and randomized subsampling was used to test for significant differences between intergenic distances for effectors versus all other genes [52]. Distances between other genomic features were calculated with the <closest> function of BEDTools (2.27.1 [53]), and hexbin plots were generated with R [54] using the geom_hex function of ggplot2. Other statistical tests were performed with R: binomial tests using binom.test, Welch’s two-sample t-tests using t.test, Spearman rank correlation tests using cor.test and non-parametric runs tests using the runs.test function of the randtests library.

## Results

### Gapless, chromosome-level genome assemblies

Long-read and short-read sequence data were generated for five strains of *Alternaria*, representing four different species in *Alternaria* section *Alternaria* (Tables 1 and S1). An average of 2.74 million Nanopore reads were produced for each strain, resulting in 20.75 Gb of raw sequence per strain and an average fold-sequence coverage of 614X. The average Nanopore read N50 was 12.99 kb, indicating that our DNA extraction and sequencing protocols resulted in sufficiently long reads for *de novo* genome assembly. For genome polishing, an average of 29.40 million Illumina short reads were used for each strain. Our assembly pipeline successfully produced gapless, chromosome-level genome assemblies for all five strains, each containing ten core chromosomes (Tables S2 and S3 and Text S4).

Literature and public sequence repositories (NCBI and JGI MycoCosm) were searched for existing section *Alternaria* assemblies that were gapless with complete chromosomes. Seven of such genomes were recovered, but they were all from only a single species, *A. alternata*. In total, our analyses included 12 gapless genomes from *Alternaria* section *Alternaria*, all of which had BUSCO completeness scores of 98.8% or greater (Table 1).

### Macrosynteny and chromosomal homology relationships

**Fig. 1.**
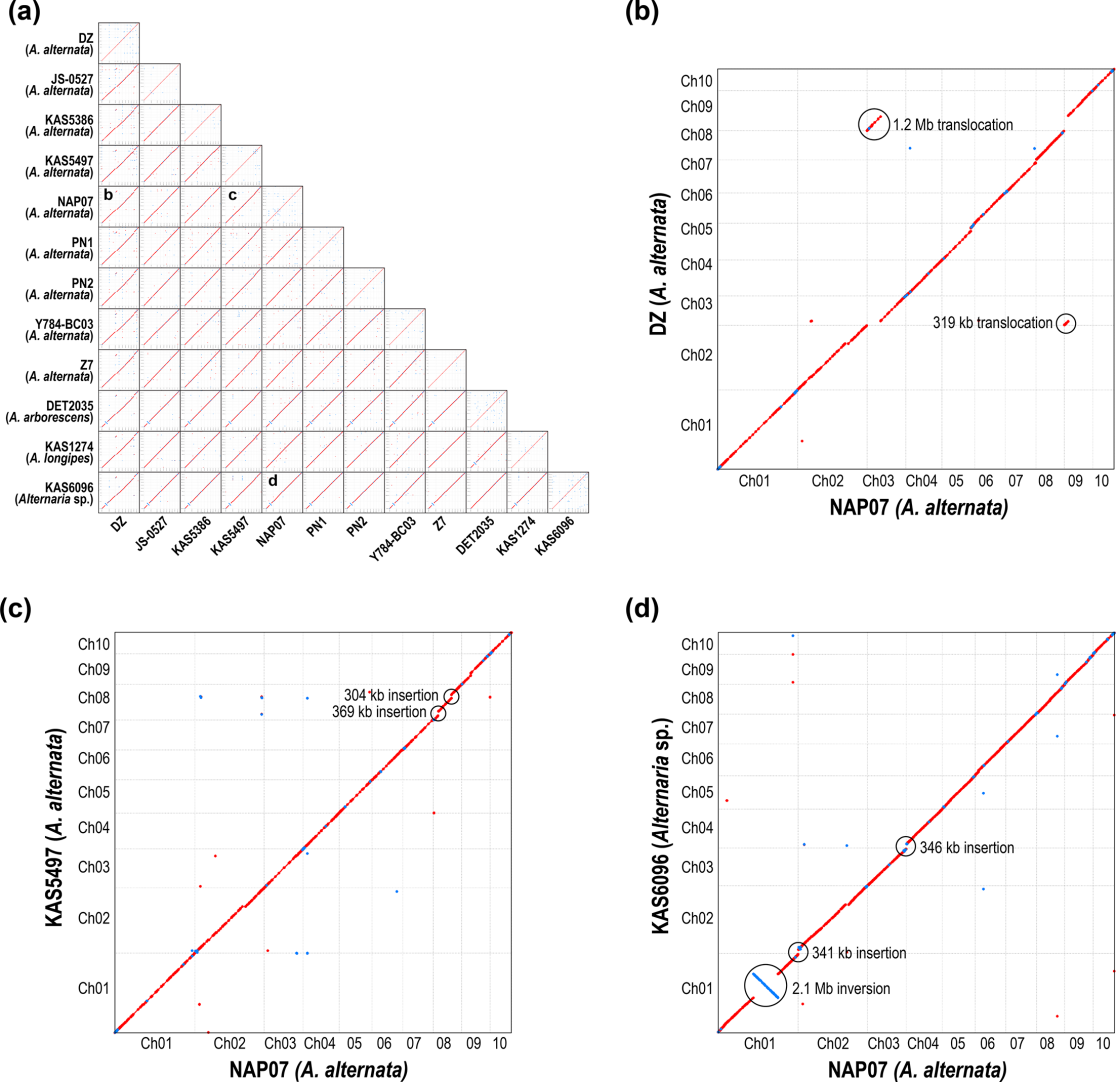
Syntenic dotplots showing general macrosynteny for pairwise comparisons of genomes. Red and blue dots indicate minimal unique matches in the same and reverse orientation, respectively. (**a**) Thumbnails of dotplots for all pairwise and self-comparisons. Higher resolution images are shown in Fig. S1. For each comparison, the reference genome is listed along the left side of the matrix. For each dotplot, the reference genome is plotted along the x-axis. Letters indicate dotplots expanded in the following figure panels to highlight the few observed structural differences. (**b**) DZ versus NAP07 (reference). (**c**) KAS5497 versus NAP07 (reference). (**d**) KAS6096 versus NAP07 (reference). For panels (b–d), large-scale structural differences are circled.

Comparison of chromosome sequences for all possible reciprocal pairwise combinations of genomes revealed the chromosomal homology relationships. To establish a unified naming convention, chromosomal nomenclature follows that of NAP07, the first gapless assembly released for *A. alternata* [31]. Some chromosomes were reverse-complemented so that all chromosomes had the same orientation and sequence directionality (Table S4). Overall, high levels of macrosynteny and structural conservation were observed. Numerous small differences were observed between genomes, but here, we focus on large-scale differences only. A visual summary of the comparative chromosomal architecture of all 66 pairwise and 12 self-comparisons is shown in Figs 1(a) and S1. Self-comparisons (diagonal of matrix, Fig. 1a) found no evidence for large intra- or inter-chromosomal duplications within genomes. Some instances of large-scale structural differences between genomes were identified (Fig. 1b–d):

1. Ch01 – A large 2.10 Mb inversion, centred around the centromere, was the only rearrangement shared between multiple strains (DET2035 and KAS6096).
2. Ch02 – A 341 kb section was added to one end in KAS6096, and a 282 kb internal insertion was found in NAP07.
3. Ch03 – 319 kb from the end of Ch09 was translocated to the end of Ch03 in DZ.
4. Ch04 – A 346 kb section was added to one end in KAS6096.
5. Ch08 – Two internal insertions of 369 and 304 kb were found in KAS5497.
6. Ch09 – 1.20 Mb from the end of Ch03 was translocated to the end of Ch09 in DZ.

These structural differences were investigated further using alternative assembly methods and raw read mapping across putative breakpoints (Text S5), and no evidence for assembly artefacts was found.

### Relationships among taxa

**Fig. 2.**
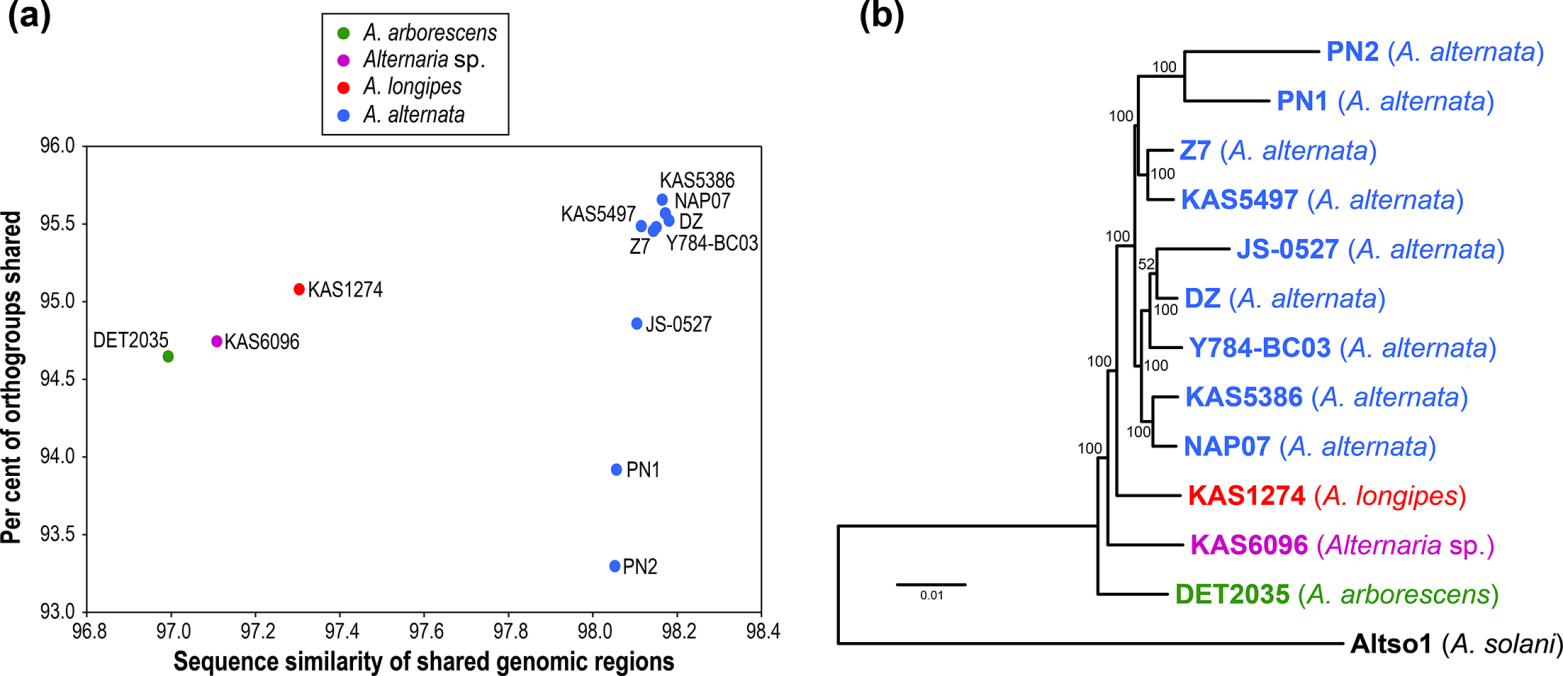
**(a**) Per cent of orthogroups shared between genomes compared to the per cent sequence similarity of all genomic regions shared between genomes. Each data point is the average for each focal strain versus all others. (**b**) Maximum likelihood phylogeny constructed from protein sequences of the 7,734 single-copy orthogroups shared by all 13 taxa. Bootstrap percentages are indicated on main branches.

Relationships among taxa were estimated in three ways:

1. Similarity at the genome sequence level (Figs 2a and S1) – The average sequence similarity of all genomic regions shared between two strains was 97.88%. The nine *A. alternata* strains were all over 98% similar to each other (average 98.47%). The other three species were more divergent, with DET2035 (*A. arborescens*) having the lowest average similarity with others (96.99%).
2. Shared genes at the orthogroup level (Figs 2a and S1) – The average percentage of orthogroups shared between genomes was 94.98%. The PN1 and PN2 strains, which belonged to *A. alternata*, had the lowest average levels of orthogroup overlap with other strains.
3. Phylogenomic approach (Fig. 2b) – A maximum likelihood phylogeny was generated from the 7,734 single-copy orthogroups shared by the 12 ingroup strains and an *A. solani* outgroup strain. As expected, the nine *A. alternata* strains formed a well-supported monophyletic group. *A. arborescens* DET2035 was the earliest diverging, followed by the undescribed species KAS6096, then *A. longipes* KAS1274.

### Consensus genomic features and chromosome architecture for *Alternaria* section *Alternaria*

#### Chromosome lengths

**Fig. 3.**
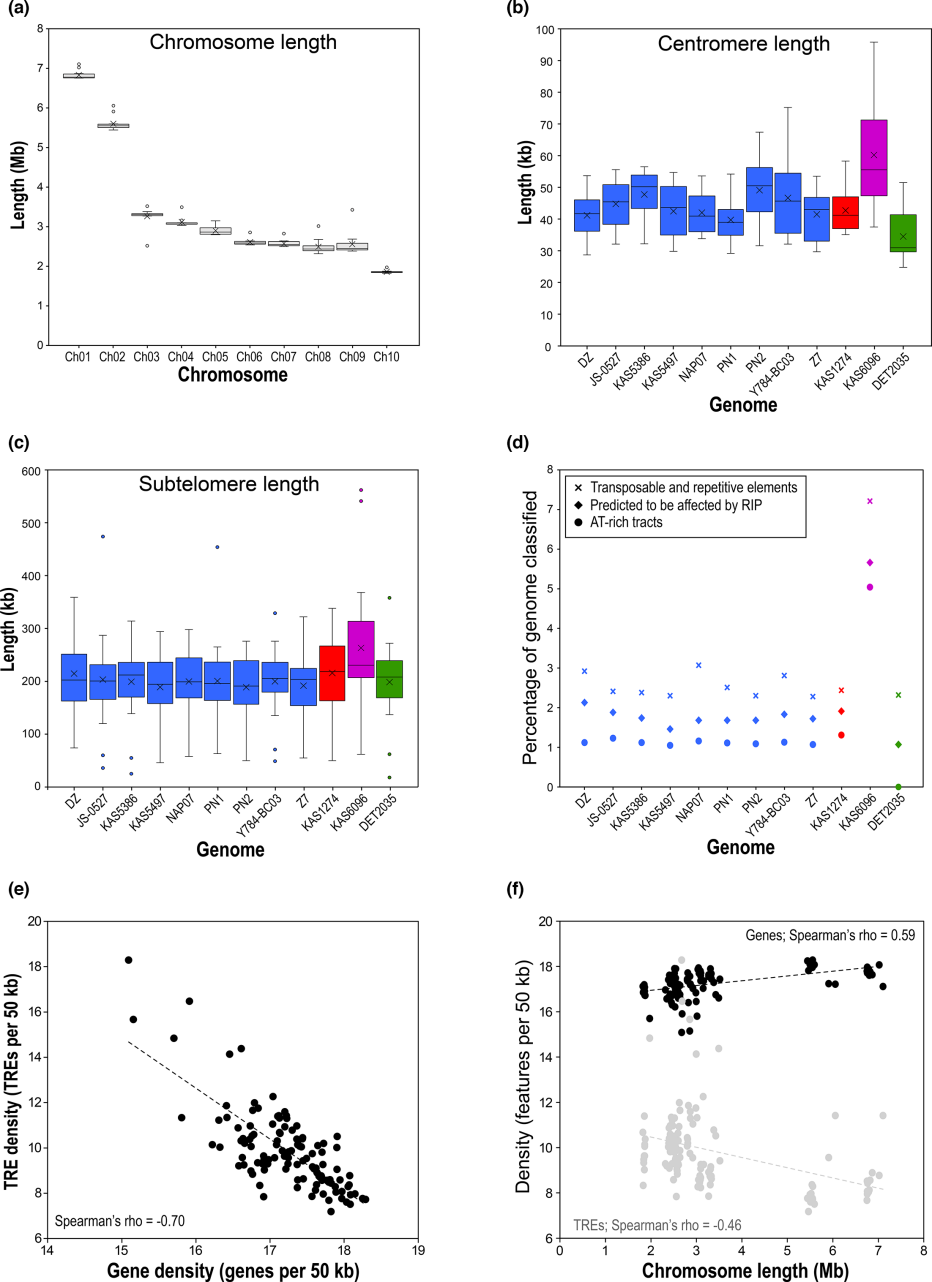
**(a**) Boxplot of chromosome lengths (*n*=12) for each of the ten homologous chromosomes. (**b**) Boxplot of centromere lengths (*n*=10) for each of the 12 genomes. (**c**) Boxplot of subtelomere lengths (*n*=20) for each of the 12 genomes. For (a–c), boxes represent the interquartile range (IQR), whiskers extend 1.5×IQR, middle line represents the median, cross represents the mean, and dots represent outlier points. (**d**) Percentages of the genome that were classified as TREs, affected by RIP mutation, and within AT-rich tracts, for each of the 12 genomes. (**e**) Scatterplot of TRE density versus gene density, for each of the 120 individual chromosomes (*P*<2.2×10^–16^). (**f**) Scatterplot of TRE density (grey, *P*<2.5×10^–7^) and gene density (black, *P*<2.2×10^–16^) versus chromosome length, for each of the 120 individual chromosomes. Colour-coding in (b–d) matches that in Fig. 2.

Mean lengths of the ten core chromosomes ranged from 6.83 to 1.86 Mb for Ch01 and Ch10, respectively (Fig. 3a and Table S5). Other than the few structural changes described above, chromosome lengths had fairly low variability across genomes. Individual chromosome lengths deviated from their respective homologue means by an average of only 2.98%.

#### Centromeres

Centromeres were an average of 44 kb in length (Fig. 3b) and were characteristically AT-rich, with GC contents of only 22–28%. The location of centromeres was typically *not* at the centre of the chromosome: the longer arm was, on average, 4.17 times longer than the shorter arm (acrocentric or submetacentric).

#### Telomeres and subtelomeric regions

Canonical telomeric repeats, averaging 22 repeats per telomere, were observed for the majority of the 120 individual chromosomes (10 chromosomes × 12 genomes; Table S5). Telomeres were identified on both ends of 72.5% (87 out of 120) of individual chromosomes. Five individual chromosomes were lacking both telomeric end repeats, but alignment to homologous chromosomes revealed that they were near full-length. On average, the recovery rate of telomeric sequences was lowest for Ch07, which can be explained by the presence of rDNA array repeats at the right terminus. The genome for DET2035 *A. arborescens* had the lowest number of identified telomeres.

Comparative genomics was used to define ‘subtelomeres’ as telomere-adjacent regions of chromosomes where increased frequencies of structural changes such as deletions, insertions and/or inversions occur. Homologous chromosomes were aligned, and the subtelomere boundary was demarcated by the breakdown of local synteny conservation (Text S6; Fig. S2). Applying this approach to the 240 analysed chromosome ends, subtelomeres comprised the terminal 18–562 kb of chromosomes (Fig. 3c; Table S6). The overall average subtelomeric length was 205 kb, which corresponds to an average of the terminal 6.79% of the chromosome on each end.

#### GC content and repeat-induced point (RIP) mutations

GC content was remarkably consistent across genomes (mean=50.94; Table 1), varying by less than 1% in terms of mean GC%. Genome-wide scans detected very limited amounts of GC bias, with an average of only 1.37% of each genome within AT-rich tracts (Fig. 3d). GC content plotted along the lengths of chromosomes showed that these AT-rich tracts typically corresponded to centromeric regions and only occasionally to short interstitial regions. The KAS6096 genome had the highest percentage of AT-rich regions (5.04%), and correspondingly, the lowest genome-wide GC% (50.10%). Predicted RIP-affected regions comprised an average of only 2.04% of each genome (Fig. 3d). Again, regions with RIP signatures typically overlapped with the AT-rich centromeres. Therefore, RIP mutation activity is not a major driver of genomic GC composition in these fungi.

#### Gene density

**Fig. 4.**
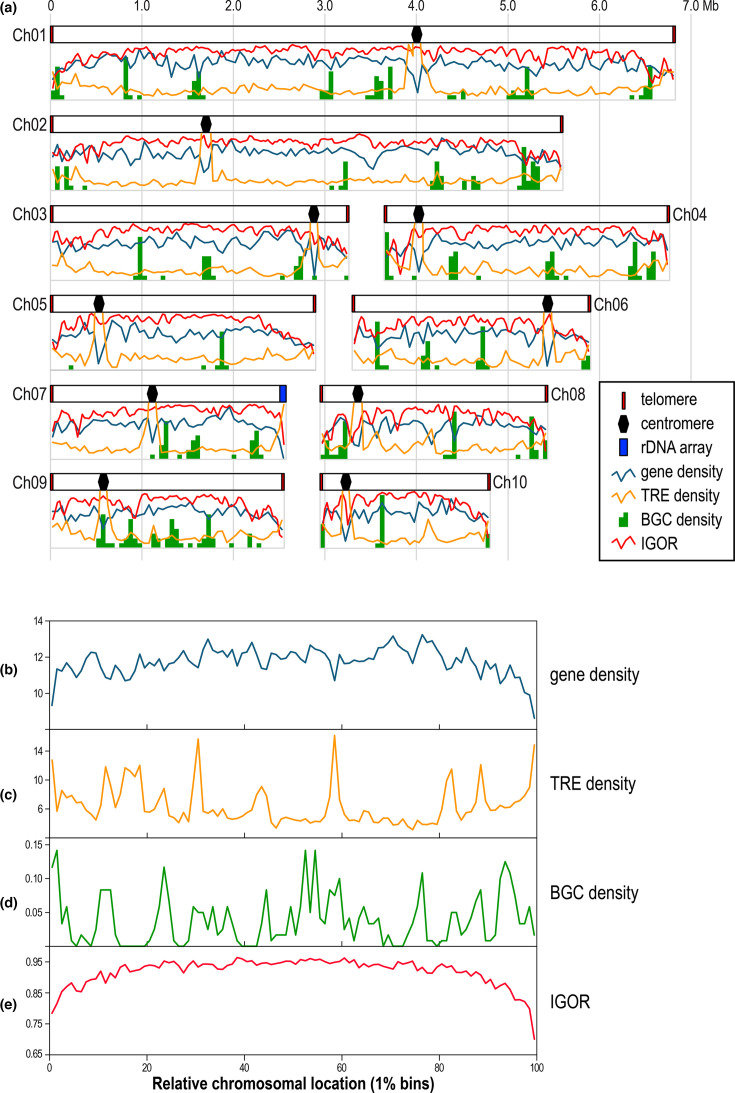
**(a**) Consensus chromosome features of *Alternaria* section *Alternaria* genomes. Displayed features/values for each chromosome are averages from all 12 genomes combined. Blue plots are mean number of genes per 50 kb interval, with a y-axis range of 5–25. Orange plots are mean number of TREs per 50 kb internal, with a y-axis range of 0–40. Green bars are mean number of secondary metabolite BGCs per 50 kb interval, with a y-axis range of 0–1.0. Red plots are mean IGOR values for 50 kb sliding windows (25 kb offsets), with a y-axis range of 0.6–1.0. Telomeres, centromeres and the rDNA array are not drawn to scale. (**b**) Mean gene density, (**c**) mean TRE density, (**d**) mean BGC density and (**e**) mean IGOR. For (b–e), displayed features/values are averages from all 120 individual chromosomes combined (12 genomes×10 chromosomes). Chromosomes were standardized by relative chromosomal locations using 1% length-equivalent bins.

Gene density was fairly similar for the different chromosomes, with lowest and highest averages being from Ch08 (16.34 per 50 kb) and Ch02 (17.93 per 50 kb), respectively (Table S7). Plotting mean gene density along the length of chromosomes (Fig. 4a, dark blue graph) revealed that, as expected, centromeric regions had lower gene density. Gene density tended to decline near chromosome ends as well, particularly for short chromosome arms with low distances between centromeres and telomeres (e.g. right arm of Ch03). This pattern often extended far beyond the ~205 kb subtelomeric regions. To test the hypothesis of biased feature distribution, data were combined for all 120 individual chromosomes. To do so, each chromosome was divided into 1% length-equivalent bins to allow standardization by relative chromosomal location. The terminal 20% of chromosomes contained proportionally less genes than central regions of the chromosomes (binomial test, *P*<2.2×10^−16^, Fig. 4b). This pattern held true when the terminal regions were expanded to the last 30% of the chromosomes (*P*<2.2×10^−16^).

#### Transposable and repetitive elements (TREs)

Very low proportions of these genomes were composed of repetitive DNA or transposable elements, with an average of only 2.93%. The KAS6096 genome had the highest percentage of TREs (7.21%), consistent with its slightly higher percentages of AT-rich tracts (Fig. 3d). For all genomes, the most abundant DNA-based transposon was the *pogo* superfamily in the Tc1/mariner class (mean=0.51%), and the most abundant LTR retroelements were gypsy-like retrotransposons (mean=0.80%). Overall, the mean number of TREs per 50 kb was 9.88, with the highest density observed for Ch08 (11.11 TREs per 50 kb; Table S7). The distribution of the number of TREs along the length of chromosomes was also determined (Fig. 4a, orange graph). As expected, TRE density was generally inversely related to gene density, with TRE density increasing drastically near centromeres and slightly near chromosome ends. For all 120 chromosomes combined, the terminal 20% or 30% contained proportionally more TREs than central regions of the chromosomes (binomial test, *P*<2.2×10^−16^ for both, Fig. 4c).

#### Biosynthetic gene clusters (BGCs)

The mean number of secondary metabolite BGCs per Mb was 1.15, with the highest density observed for Ch09 (1.92 BGCs per Mb; Table S7). Ch05 was particularly low in BGC density, with only 0.35 BGCs per Mb. Localization of BGC density in 50 kb windows (Fig. 4a, green bars) revealed that the distribution of BGCs was highly uneven along chromosomes. For all 120 chromosomes combined, the terminal 20% or 30% did not contain significantly more BGCs than central regions of the chromosomes (binomial test, 0.29<*P*<0.34 for both, Fig. 4d). In general, BGC hotspots were not clustered in particular chromosomal regions.

#### Inter-genomic orthogroup retention (IGOR)

We calculated a gene-based metric called IGOR, defined as the proportion of other genomes that possess a homologue (orthogroup member) of a focal gene. An IGOR value of 1.0 indicates maximum retention, meaning that a homologue of the focal gene was identified in 100% of the other genomes. An IGOR value of 0 indicates that the focal gene has no homologues in any other genomes. Note that IGOR is independent of gene density because it is calculated on a per-gene basis. IGOR was calculated for every gene in each of the 12 genomes, and mean IGOR along the length of chromosomes was plotted (Fig. 4a, red graph). A strong pattern of IGOR reduction for genes located in regions proximal to the chromosome ends was observed, the effect of which could extend beyond the ~205 kb subtelomeric regions. When the relative patterns were combined for all 120 chromosomes (Fig. 4e), IGOR values had a clear tendency to decrease gradually near the ends of chromosomes. Genes located in the terminal 20% regions had significantly lower mean IGOR values than genes in central regions (terminal=0.88; central=0.94; t-test, *P*<2.2×10^−16^).

### Accessory chromosomes and regions

Only one of our five newly generated genomes possessed scaffolds that could not be assigned to core chromosomes. For KAS5386, two unplaced scaffolds with lengths of 510.93 and 319.43 kb did not show homology to any core chromosomes, and each possessed 18–21 canonical telomeric repeats on only one end. The core chromosome complement of KAS5386 was already complete, with identified telomeres on both ends of all chromosomes (except the rDNA-bearing end of Ch07), so these unplaced scaffolds were classified as ‘accessory’.

Comparisons with other genomes revealed that ~80% of the 510.93 kb accessory scaffold from KAS5386 was homologous to a putative accessory chromosome from NAP07. Interestingly, a putative accessory chromosome from Z7 also shared the same regions of homology (Fig. S3). Annotation of the 98 genes from the KAS5386 accessory scaffold identified two predicted BGCs: an uncharacterized terpene and a type 1 PKS whose backbone gene had 97.9% similarity to 6-methylsalicylic acid synthase (MSAS). The MSAS BGC is commonly found on accessory chromosomes [55], providing further support for their accessory status. The KAS5386 accessory scaffolds did not harbour any of the previously described host-specific toxin (HST) BGCs that are often carried on accessory chromosomes. The absence of a shared HST-BGC is not surprising given that these three strains were sampled from peach, tangerine and a wild reed grass. This finding also demonstrates that accessory scaffolds can be shared between strains even if they lack any obvious pathogenicity or virulence factors. These accessory chromosomes were not included in any of the other calculations presented here.

The five large-scale insertions present in KAS5497, KAS6096 and NAP07 also represent accessory genomic regions. These insertions had an average of 10.29 genes per 50 kb, which is only 59.2% of the overall average for all core chromosomes (17.38 per 50 kb). High-level functional annotation of the 338 included genes revealed that ‘secondary metabolites biosynthesis, transport and catabolism’ was the most common COG (Clusters of Orthologous Groups) category, followed by ‘lipid transport and metabolism’ (Fig. S4). Inspection of detailed gene functions in the former COG category found an abundance of oxidoreductases, such as dehydrogenases and monooxygenases. The latter COG category was predominantly hydrolases such as phospholipases. No BGCs were predicted in these accessory regions, and no genes with obvious roles in pathogenicity or virulence were found.

### Correlations between features of 120 individual chromosomes

With a sample size of 120 assembled chromosomes, we tested for correlations between chromosome length, gene density, TRE density, BGC density and mean IGOR. Most pairwise combinations of these five features were significantly correlated, either positively or negatively (Table 2). The strongest correlation (Spearman rho=−0.70) was a negative relationship between gene density and TRE density (Fig. 3e). Genes and TREs both occupy the finite amount of chromosomal space, often in a mutually exclusive manner, so this inverse correlation could be expected. Interestingly, chromosome length was significantly correlated with gene and TRE density (Fig. 3f). Of course, longer chromosomes are expected to have a greater number of genes in total, but we found that longer chromosomes tended to be more gene-dense and less TRE-dense (per unit length). Mean IGOR was also positively and negatively correlated with gene density and TRE density, respectively. This pattern suggests that chromosomes with greater densities of TREs are more prone to small-scale structural changes that cause the gain/loss of genes (i.e. reduced IGOR).

**Table 2.** Correlations between genome features at the chromosome level (*n*=120). Values are Spearman rank correlation (rho), with *P*-values in brackets

	Chromosome length	Gene density	TRE density	BGC density
**Gene density**	0.59 (*P*<2.2×10^-16^)			
**TRE density**	−0.46 (*P*<2.5×10^-7^)	−0.70 (*P*<2.2×10^-16^)		
**BGC density**	−0.26 (*P*<0.005)	−0.18 (*P*<0.05)	0.04 (*P*>0.63)	
**Mean IGOR**	0.25 (*P*<0.006)	0.37 (*P*<4.0×10^-5^)	−0.27 (*P*<0.003)	−0.21 (*P*<0.025)

### Bipartite genome compartmentalization

**Fig. 5.**
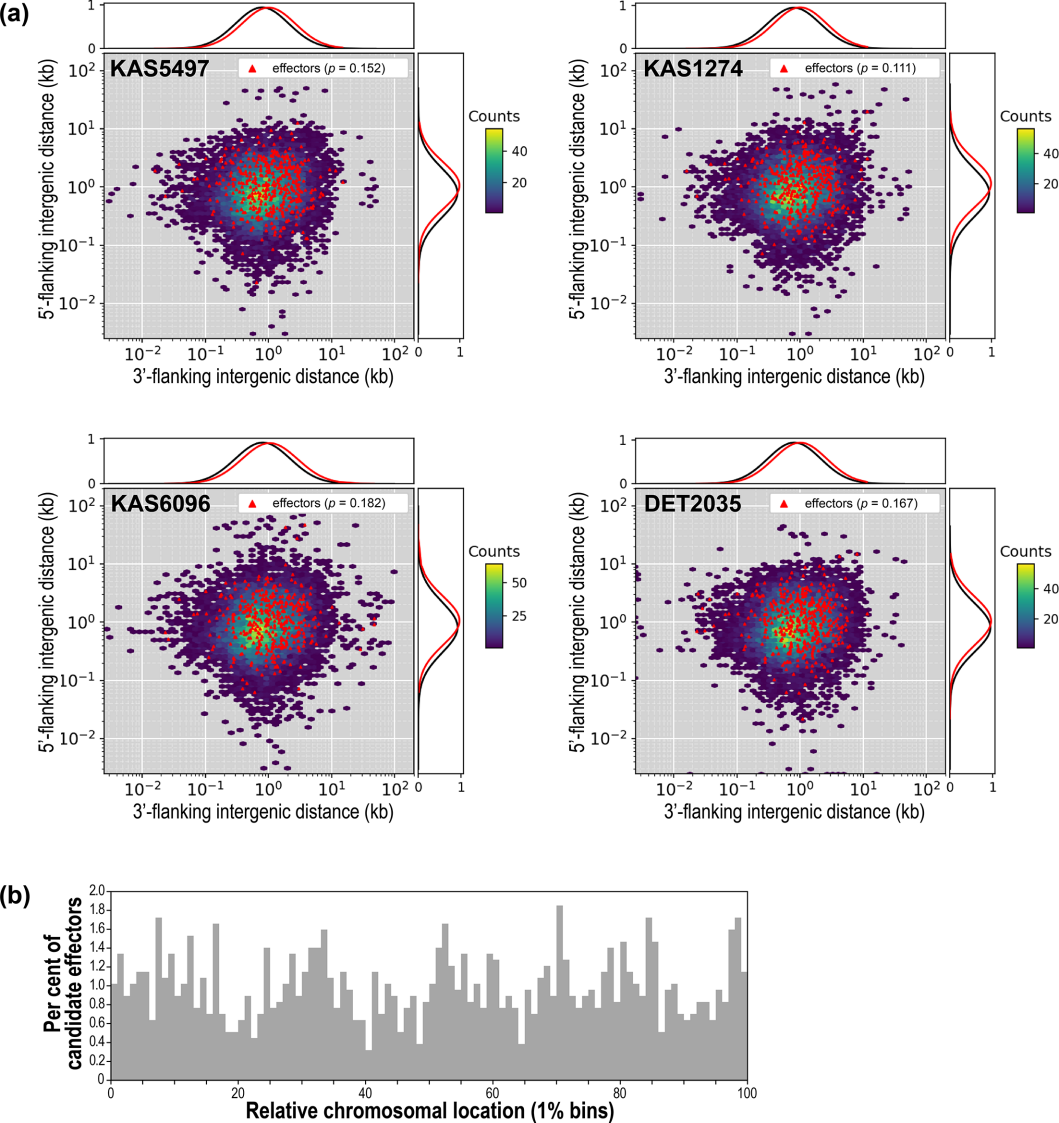
**(a**) Hexbin plots of gene density as a function of 5′- and 3′-flanking intergenic distances, for four representative genomes. Hexbin colour (right legend) indicates the gene count within each hexbin. Red triangle markers indicate putative effector genes. Line graphs show the frequency distributions of effectors (red) and all other genes (black). The *P*-values are from tests to determine if effector intergenic distances were different from those of all other genes. (**b**) Chromosomal locations of candidate effector genes, for four representative genomes combined. Grey bars show the per cent of effectors that are located within the chromosomal location bin (x-axis).

Representative genomes were searched for evidence of bipartite genome compartmentalization (two-speed genome). Using the standard test employed for pathogenic fungi [15,16], we investigated if candidate effector genes displayed biased localization to gene-sparse regions of the genome (Fig. 5a, Table S8). For each representative genome, the flanking intergenic distances for effector genes were not significantly different from those of all other genes (0.11<*P*<0.19 for each). In other words, effectors were not disproportionately found in gene-poor regions, a result inconsistent with the two-speed genome model. Next, we tested if candidate effectors were localized to chromosome ends, which we have shown to be highly dynamic genomic regions. The terminal 20% or 30% of chromosomes did not contain significantly more effectors than central regions of the chromosomes (binomial test, 0.21<*P*<0.32 for both, Fig. 5b).

**Fig. 6.**
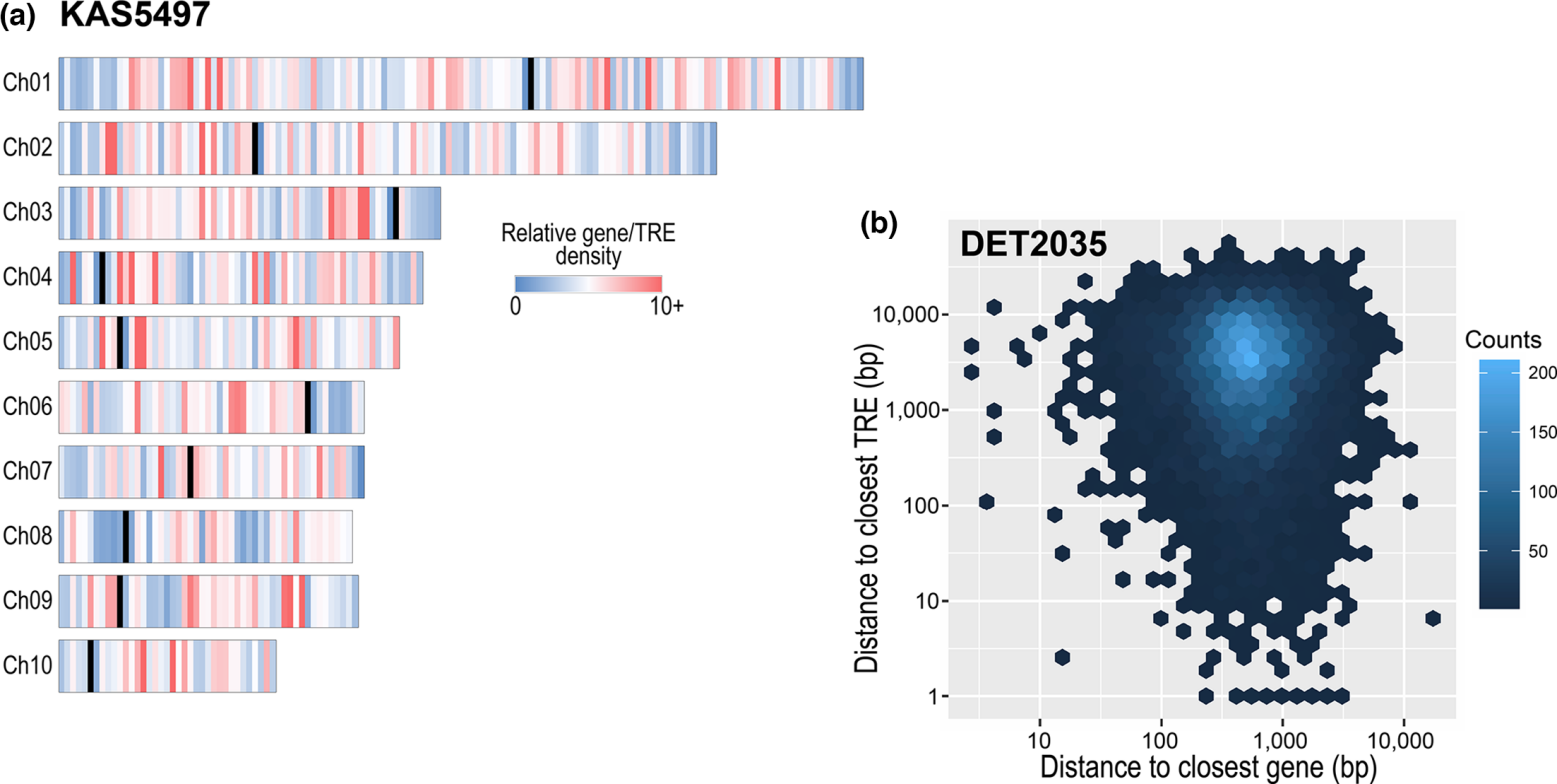
**(a**) Relative gene/TRE densities for the KAS5497 genome, in 50 kb intervals along each chromosome. Colour (inset legend) of vertical bars indicates relative densities, and black bars indicate the location of centromeres. (**b**) Hexbin plot of gene density as a function of distance to closest gene and distance to closest TRE, for all genes in the DET2035 genome. Hexbin colour (right legend) indicates the gene count within each hexbin.

We examined, in more detail, the physical properties of genomes that are essential components of bipartite genome compartmentalization. First, the low proportion of AT-rich tracts in these genomes (Fig. 3d), with tight unimodal GC% distributions around the genomic means (Fig. S5), indicated that alternating GC-rich/AT-rich isochores did not exist in these genomes. Second, plots of distances between genes (background plots in Fig. 5a) provided no evidence for distinct gene-dense and gene-poor regions. The distribution of distances between neighbouring genes did not form multiple clusters, indicating a single-compartment genome. Third, we explored if genomes were actually divided into gene-rich/TRE-poor versus gene-poor/TRE-rich regions. The extremely low prevalence of TREs in these *Alternaria* genomes already suggested that this was not the case, but plots of relative gene and TRE density along 50 kb windows confirmed this conclusion (example of KAS5497 in Fig. 6a). While there is clear variation in relative gene/TRE densities along chromosomes, long stretches of consistently high or low relative gene/TRE densities were uncommon. The only detectable pattern was lower relative gene/TRE densities proximal to centromeres and telomeres, as previously observed. Excluding the 200 kb terminal regions, non-parametric runs tests generally supported the random distribution of gene/TRE densities along chromosomes. For example, only two of the ten KAS5497 chromosomes showed marginal evidence of non-randomness (Ch01, *P*<0.031; Ch09, *P*<0.008).

Finally, the distances between a focal gene and the closest gene, and the closest TRE, failed to support compartmentalization (example of DET2035 in Fig. 6b). With physically compartmentalized genomes, genes in gene-rich/TRE-poor regions would be close to other genes but far from TREs, whereas genes in gene-poor/TRE-rich regions would be far from other genes and close to TREs. This pattern of a bimodal distribution in opposing quadrants of the graph was not observed. Furthermore, distances between a focal TRE and the closest TRE or gene also indicated a non-clustered distribution of TREs. In fact, the mean distance between a TRE and the closest other TRE was significantly greater than the mean distance between a TRE and the closest gene (DET2035 means of 2,669.8 and 2,327.5 bp, respectively; t-test, *P*<3.54×10^−6^).

## Discussion

Here, we take advantage of the exceptional power of resolution provided by gapless assemblies to study the evolution of genomic and chromosomal architecture in *Alternaria* section *Alternaria*. Gapless assemblies have more complete recovery of gene content, repetitive or duplicated regions and transposable elements [3,4], and the chromosomal architecture with locations and orientations of all genomic features can be determined accurately. We did not perform detailed analyses of gene ontology, functional pathways or transposable element classifications, as these have already been explored in previous reports [30,33]. Rather, we focused on large-scale physical attributes of genomic organization, or aspects that can only be studied using chromosome-level genome assemblies. Nine of the analysed genomes were from *A. alternata*, the main species for much of *Alternaria* research, but we also generated the first gapless assemblies for three additional species. Restricting our analyses to individuals within the same or closely related species reveals the changes in genomic architecture that occur over short evolutionary timeframes. This baseline can then be used to interpret broader comparisons made at successively greater levels of evolutionary divergence. Indeed, we view this study as the starting point for future research that continually expands in taxonomic breadth to incorporate gapless genomes from multiple other sections of *Alternaria*, and eventually, other closely related genera in the *Pleosporaceae* family.

Core chromosome structure is fairly well conserved in *Alternaria* section *Alternaria*. At the highest level, a stable set of ten core chromosomes has been maintained over the evolutionary history of these four *Alternaria* species. Lengths of chromosome homologues had low variability across genomes, and large-scale structural changes were rare and typically involved only a few hundred kilobases of sequence (Fig. 1). Comparisons of the nine genomes from the species *A. alternata* revealed only five significant rearrangement events in total, and only one genome had a large inter-chromosomal translocation.

The conservation of chromosomal architecture within a single species has been studied in other Ascomycete fungi, with mixed findings. Similar to that observed here in *Alternaria*, general macrosynteny was reported for *Aspergillus fumigatus* [56], *Cladosporium fulvum* [12], *Fusarium circinatum* [10], *Parastagonospora nodorum* [57] and *Tolypocladium inflatum* [58]. These studies found mostly macrosyntenic core chromosomes with only a few major rearrangements, and inter-chromosomal translocations were rare. In contrast, multiple large-scale rearrangements, both intra- and inter-chromosomal, were commonly found between strains of *Claviceps purpurea* [59], *Cordyceps militaris* [14], *Pyrenophora tritici-repentis* [24] and *Verticillium dahliae* [8,9].

Several factors could be causing these differences in observed levels of intra-specific macrosynteny. First, studies could be sampling different levels of intra-specific biodiversity (e.g. local versus global). Similarly, reduced effective population sizes caused by recent population bottlenecks or strong selective sweeps are expected to reduce chromosomal diversity. Neither of these is a likely explanation for the low chromosomal diversity in *A. alternata*: it is considered a globally widespread, genetically diverse species [29] and the strains analysed here were collected from eight different plant hosts in five countries. Another potential factor is differences in rates of sexual reproduction. Large structural differences in chromosomal homologues may cause misalignment problems at metaphase pairing during meiosis. Such mispairing can disrupt proper meiotic processes, leading to aneuploidy, meiotic arrest and non-viable progeny [60]. Thus, in sexually reproducing species, there is strong purifying selection for the conservation and homogenization of chromosomal structure across individuals. In species that reproduce by asexual means only, chromosomal architecture may be free to vary because there is no requirement for pairing of homologous chromosomes at meiosis. This chromosomal drift has been proposed as the explanation for accumulation of chromosomal diversity in the strictly asexual *V. dahliae* [9]; however, *C. fulvum* [12] and *A. alternata* have no known sexual cycle but still display high macrosynteny. Do these putatively asexual species actually have a cryptic sexual cycle [12,29], which occurs frequently enough for purifying selection to remove large-scale chromosomal outliers from the population? Or are these species strictly asexual and some other form of selection is maintaining chromosomal macrosynteny?

The pattern of general macrosynteny held true even as comparisons were made between successively more divergent *Alternaria* species, and the expected gradual stepwise accumulation of large-scale rearrangements through time was not observed. For example, the *A. arborescens* DET2035 strain was the most divergent (Fig. 2), but it displayed only one large-scale difference against most other strains (Fig. 1a): the 2.10 Mb centromere-spanning inversion in Ch01 shared with the undescribed species KAS6096. Based on phylogeny tracking (Fig. 2), this configuration likely represents the ancestral state, with the actual inversion occurring only once and being inherited by *A. longipes* and *A. alternata*. For section *Alternaria*, the assumption of syntenic placement and orientation of contigs in fragmented draft genomes holds true in general, when comparing closely related strains and species. With future work, it will be interesting to determine at what divergence level this conserved synteny starts to break down. In contrast to *Alternaria*, large-scale inter-chromosomal rearrangements are extremely common between species within other genera, such as *Trichoderma* [61], *Epichloë* [62] and *Verticillium* [63]. The high frequency of inter-chromosomal reshuffling within these genera raises the question of how mesosynteny – the conservation of gene content within homologous chromosomes, without conservation of gene order and orientation – can be maintained across long evolutionary time frames, as observed between some groups of filamentous Ascomycetes [64]. Comparative analysis of an evolutionary transect of appropriately chosen fungal groups could help to determine the levels of phylogenetic divergence at which transitions from macrosynteny to microsynteny, and potentially mesosynteny, occur.

The physical aspects of genomic architecture were compared across 12 strains, establishing the consensus characteristics for a typical *Alternaria* section *Alternaria* genome. As expected, variation was generally lower for within-species comparisons than for between-species comparisons. All genomes had quite similar values for most features (Table 1, Fig. 3), with the one exception being the genome of KAS6096 (undescribed *Alternaria* species). The KAS6096 genome was the largest and had the longest centromeres and subtelomeres, the lowest GC content and the greatest proportion of TREs. These patterns can be explained because these features are not independent of each other. For example, the proliferation of TREs is associated with expansions in genome size [65], and major differences in genome size between closely related fungi can often be accounted for in repetitive DNA content alone. Since centromeres and subtelomeres are typically quite repetitive, they would also increase in size as a result of TRE proliferation. Finally, TRE-rich regions are correlated with reduced GC content [43], so overall decreases in mean GC%, and increases in observable AT-rich regions, would also be expected.

Subtelomeres are often associated with increased rates of mutation, recombination and gene turnover, suggesting that they are hotbeds for genomic evolution and innovation [66,68]. Subtelomeres are simply regions that are immediately adjacent to the telomeres on the chromosome ends, but there is no clear agreement on how they should be delineated (e.g. pre-determined length; gene density decline [69]). Here, we analysed full-chromosome alignments to determine where increased frequencies of moderately sized structural changes (indels and inversions) began to occur and used this breakdown of colinearity to demarcate the subtelomere boundary. Applying this method to our sample of 240 chromosome ends resulted in an estimated mean subtelomere length of 205 kb; however, most of our observed gene-based subtelomere-related trends (e.g. IGOR reductions) extended well beyond this distance. As such, most of our analyses of feature distributions focused on the terminal 20% of chromosomes, which corresponds to ~3X the subtelomere length.

Terminal regions of chromosomes had significantly lower gene density and higher TRE density (Figs 4 and 5a). Work on other fungi has suggested that certain classes of genes may have biased localization near rapidly evolving chromosome ends; however, we found no evidence for this pattern in section *Alternaria*. For example, work on *Aspergillus* [70,71] has suggested that secondary metabolic gene clusters may be preferentially located in subtelomeric regions, but we failed to detect such an association here (Fig. 4d), similar to the findings in *Tolypocladium* [57]. Effector genes are predicted to adaptively localize to subtelomeric regions because they benefit from increased diversification caused by the locally elevated rates of genomic plasticity [72,73]. For section *Alternaria*, we found that candidate effectors were fairly evenly distributed along the length of chromosomes (Fig. 5b). More rigorous analyses of gapless genomes from additional diverse taxa will determine if subtelomere-biased localization of specific gene classes is rare or common.

Another clear trend was that genome content variation is greater near chromosome ends, with a gradual decrease towards the more stable, central regions. Genome-wide gene turnover (gain or loss of a homologue from a genome) is elevated near chromosomal termini, as evidenced by significantly lower mean IGOR values (Fig. 4). IGOR is akin to pangenome plots (core versus accessory) but with added dimension of chromosomal location. In other words, the probability that genes or genomic regions are considered accessory (rather than core) increases towards chromosome ends. This pattern has been reported in other fungi; for example, McCarthy and Fitzpatrick [67] found that accessory genes in four model fungal species tended to be clustered in subterminal regions. Our simple IGOR metric allows for the fine-scale relative quantification of orthogroup retention over multiple genomes.

Rapid gene turnover is an inherent property of these regions of genomic plasticity and is not driven by the enrichment of certain functional classes of embedded genes [69]. The most plausible explanation for this pattern is elevated recombination frequencies and the associated gene gain/loss caused by non-allelic homologous recombination (NAHR). Studies in various biological systems have found that recombination rates tend to increase gradually towards chromosome ends and peak in subtelomeric regions [66,74, 75]. Subtelomeric regions are often rich in duplicated genes or identical copies of TREs – the substrate for NAHR – and elevated rates of intra- or inter-chromosomal NAHR can lead to numerous small-scale genomic rearrangements in those regions [76]. Even *mitotic* sister chromatid exchange has been shown to occur at highly elevated rates at chromosome ends [77], which may explain how the pattern is observed in fungi that lack a known sexual cycle. An important area that requires further investigation via more detailed mechanistic studies is the differential impacts of mitotic versus meiotic recombination on genomic variability.

Pathogens and hosts compete in a co-evolutionary arms race which may promote the rapid diversification of pathogenicity- or virulence-associated genes in the pathogen. If these genes can be physically clustered into distinct regions of the genome that are able to diversify more rapidly, without compromising the function of the rest of the genome, then such compartmentalization would be evolutionarily beneficial (two-speed, or two-compartment, model [15,17]). While the genomes of some pathogens do have bipartite structure, with distinct repeat-dense, gene-sparse regions that are enriched for effector genes (e.g. *Leptosphaeria maculans* [19], *Phytophthora infestans* [18], *Epichloë* [78] and *C. fulvum* [12,79]), further investigation of other fungal pathogens has found that the lack of compartmentalization is also quite common (e.g. *Sclerotinia sclerotiorum* [20], *Ramularia collo-cygni* [23], *P. tritici-repentis* [24], *Puccinia striiformis* [22] and *Blumeria graminis* [21]). Here, we present multiple types of analyses demonstrating that section *Alternaria* genomes do not have physical bipartite compartmentalization. We found no evidence for alternating GC-rich/AT-rich isochores, distinct gene-rich/TRE-poor versus gene-poor/TRE-rich regions or biased localization of candidate effectors.

Why do these *Alternaria* species lack genome compartmentalization? One potential explanation is that many *Alternaria* species, including *A. alternata*, have quite broad host ranges and therefore experience less pronounced ‘gene-for-gene’ interactions with their hosts [20]. With narrow host range pathogens, the co-evolutionary arms race promotes repeating cycles of diversification of host-specific virulence genes in the pathogen, in response to new resistance genes in the host. Therefore, narrow host range pathogens may experience stronger selection for genome compartmentalization. While most examples of highly compartmentalized genomes do come from narrow host range pathogens, there are clear examples from broad host range pathogens as well. For instance, *V. dahliae* can infect ~200 different plant hosts but still displays strong evidence for compartmentalization [8]. However, many of the non-compartmentalized pathogens mentioned above appear to infect only a single host species (e.g. *P. tritici-repentis* and *P. striiformis* on wheat), so host range breadth cannot be the only factor influencing genome compartmentalization.

Another potential explanation is that these *Alternaria* genomes have insufficient proportions of TREs to facilitate the compartmentalization process. As transposable elements proliferate and spread throughout the genome, they may preferentially accumulate in gene-poor regions because their insertion events are less likely to disrupt essential genes (i.e. decreased purifying selection). Genomes with low TRE content overall, or low transposable element activity, simply may not be able to compartmentalize, regardless of how strong the selection pressure may be. More studies on a wider diversity of taxa are needed to determine whether the level of observed bipartite compartmentalization is positively correlated with the genome-wide TRE density. Some examples that are inconsistent with this hypothesis already exist: *B. graminis* is extremely rich in TREs (~74% repetitive elements), but they are dispersed uniformly across the one-speed genome [21]. Transposable elements may also actively promote genome plasticity by causing DNA breaks during excision events and by forming the substrate for rearrangements by unfaithful homology-based repair [80]. In *Epichloë*, for example, large-scale structural changes appear to be driven by recombination breakages in AT-rich, repeat-dense regions [62]. Future work should investigate this potentially complex relationship between TRE content, genome compartmentalization and intra-specific macrosynteny conservation.

Other authors have already noted how the dichotomy between one- and two-speed models does not hold, acknowledging that genomes can have multiple compartments and/or speeds [16,17]. Furthermore, physical compartmentalization of a genome and the rate of evolution of those compartments are actually two separate concepts [16]. Indeed, genomes that lack formal bipartite compartmentalization can have regions that evolve at different rates, such as subtelomeres. In *Alternaria*, the gradual change in mean IGOR values along the length of the chromosomes suggests a continuum rather than classification into distinct speed classes. Our viewpoint is that all genomes have a continuum of speeds, regardless of their large-scale genomic architecture. Several overlapping and interacting factors may have an influence, such as subtelomere effects, heterochromatin versus euchromatin, core versus accessory regions, TRE distributions and activity, recombination landscape, genome defence mechanisms, pathogenic lifestyle and selection pressures. We should expect a wide spectrum of possibilities regarding compartments and associated rates of evolution, with the goal of determining which factors ultimately have the greatest impact on the final evolutionarily stabilized architecture of a genome.

## Conclusions

When comparing our results to those from other fungi, it becomes clear that patterns of fungal genomic architecture are diverse and complex (e.g. proportion of TREs, intra- and inter-specific macrosynteny, distribution of BGCs and effectors and physical bipartite compartmentalization). While specific trends have been well documented from some fungi, mainly from narrow host range plant pathogens, focusing on particular examples may have inflated the expectation of the generality of such trends. A broad comparative synthesis across the other fungal groups is needed before predictions can be made based on taxonomic position or lifestyle. Generalized conclusions can be made with confidence once a controlled, systematic comparison of large datasets of gapless genomes from strategically chosen, representative fungal taxa is performed. Studies of population-level genomic variation reveal how individuals of the same species accumulate sequence and structural differences, and how these may impact their evolutionary potential and adaptability. Genome-wide association studies can leverage this wealth of information to link important phenotypes with specific genomic variation. The gapless, chromosome-level genomes from section *Alternaria* presented here are useful resources for studying the evolutionary processes and mechanisms that generate genomic diversity, both within and between species of plant-pathogenic fungi.

## Supplementary material

10.1099/mgen.0.001686 Uncited Supplementary Material 1.

## Abbreviations

BGCs: biosynthetic gene clusters
COG: Clusters of Orthologous Groups
HST: host-specific toxin
IGOR: inter-genomic orthogroup retention
IQR: interquartile range
MSAS: 6-methylsalicylic acid synthase
MUMs: maximal unique matches
NAHR: non-allelic homologous recombination
NCBI: National Center for Biotechnology Information
NRPS: non-ribosomal peptide synthetase
PKS: polyketide synthase
RIP: repeat-induced point
TREs: transposable and repetitive elements
