# Supplementary material for: Evolution of genomic architecture of the plant-pathogenic fungus Alternaria revealed by comparative analyses of 12 chromosome-level assemblies
Source: Microb Genom. 2026 Apr 10;12(4):001686. doi: 10.1099/mgen.0.001686 (PMC13068351; doi:10.1099/mgen.0.001686)
Supplement: Uncited Supplementary Material 1. [file mgen-12-01686-s001.pdf]

Evolution of Genomic Architecture of the Plant-Pathogenic Fungus *Alternaria* Revealed by Comparative Analyses of Twelve Chromosome-Level Assemblies

Jeremy R. Dettman, Natalie E. Kim, and Kasia Dadej

SUPPPLEMENTARY MATERIAL

Includes:

SUPPLEMENTARY FIGURE S1: Genome-wide syntenic dotplots, orthogroup sharing, and percent sequence similarities.
SUPPLEMENTARY FIGURE S2: Example of subtelomere demarcation.
SUPPLEMENTARY FIGURE S3: Accessory chromosome dotplots.
SUPPLEMENTARY FIGURE S4: Accessory gene functional annotation.
SUPPLEMENTARY FIGURE S5: GC content plots.

SUPPLEMENTARY TABLE S1: Summary of sequence data outputs.
SUPPLEMENTARY TABLE S2: Versions of NECAT assemblies chosen for subsequent polishing.
SUPPLEMENTARY TABLE S3: Cross-validation of NECAT assemblies with CANU assemblies.
SUPPLEMENTARY TABLE S4: Chromosomal homology relationships.
SUPPLEMENTARY TABLE S5: Chromosome lengths and telomeric repeat presence.
SUPPLEMENTARY TABLE S6: Estimated lengths of subtelomeric regions.
SUPPLEMENTARY TABLE S7: Summary statistics for chromosomes, averaged across all twelve genomes.
SUPPLEMENTARY TABLE S8: Numbers of secreted proteins and candidate effectors.

SUPPLEMENTARY TEXT 1: Polishing assemblies.
SUPPLEMENTARY TEXT 2: Repeat annotation.
SUPPLEMENTARY TEXT 3: Calculation of inter-genomic ortholog retention (IGOR) values.
SUPPLEMENTARY TEXT 4: Manual joining of overlapping contigs for *A. alternata* KAS5386.
SUPPLEMENTARY TEXT 5: Further investigation of structural differences.
SUPPLEMENTARY TEXT 6: Demarcation of subtelomeric regions.

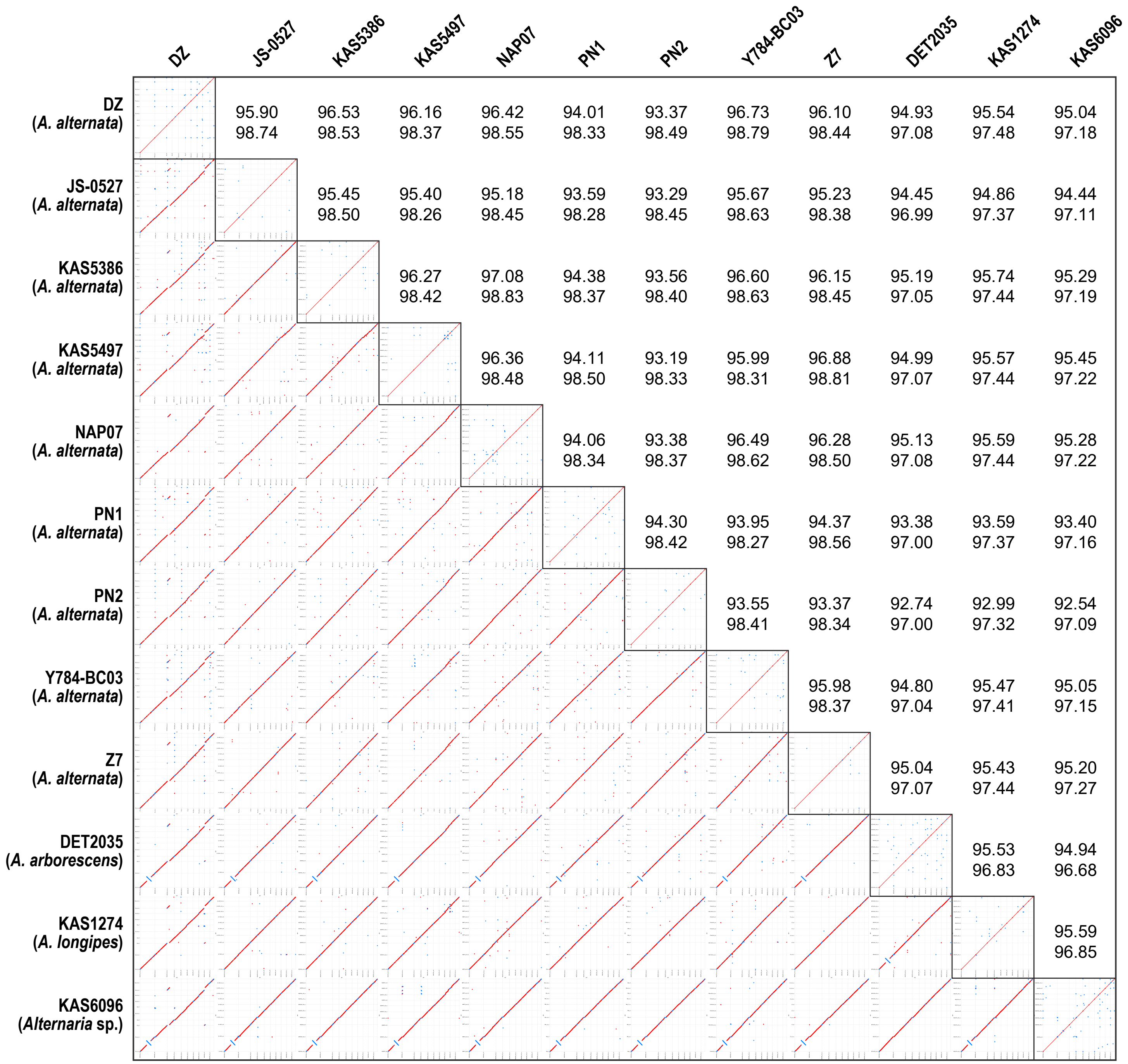

SUPPLEMENTARY FIGURE S1:
Lower left matrix: Syntenic dotplots showing general macrosynteny for pairwise comparisons of genomes. For each comparison, the reference genome is listed along the left side of the matrix. For each dotplot, the reference genome is plotted along the x-axis. Red and blue dots indicate minimum unique matches in the same and reverse orientation, respectively.

Diagonal: Syntenic dotplots for self-comparisons of genomes.

Top right matrix: For each pairwise comparison, the upper number is percentage of orthogroups overlapping between genomes, and lower number is average percent sequence similarity for all shared genomic regions. The reference genome is the genome listed along the left side of the matrix.

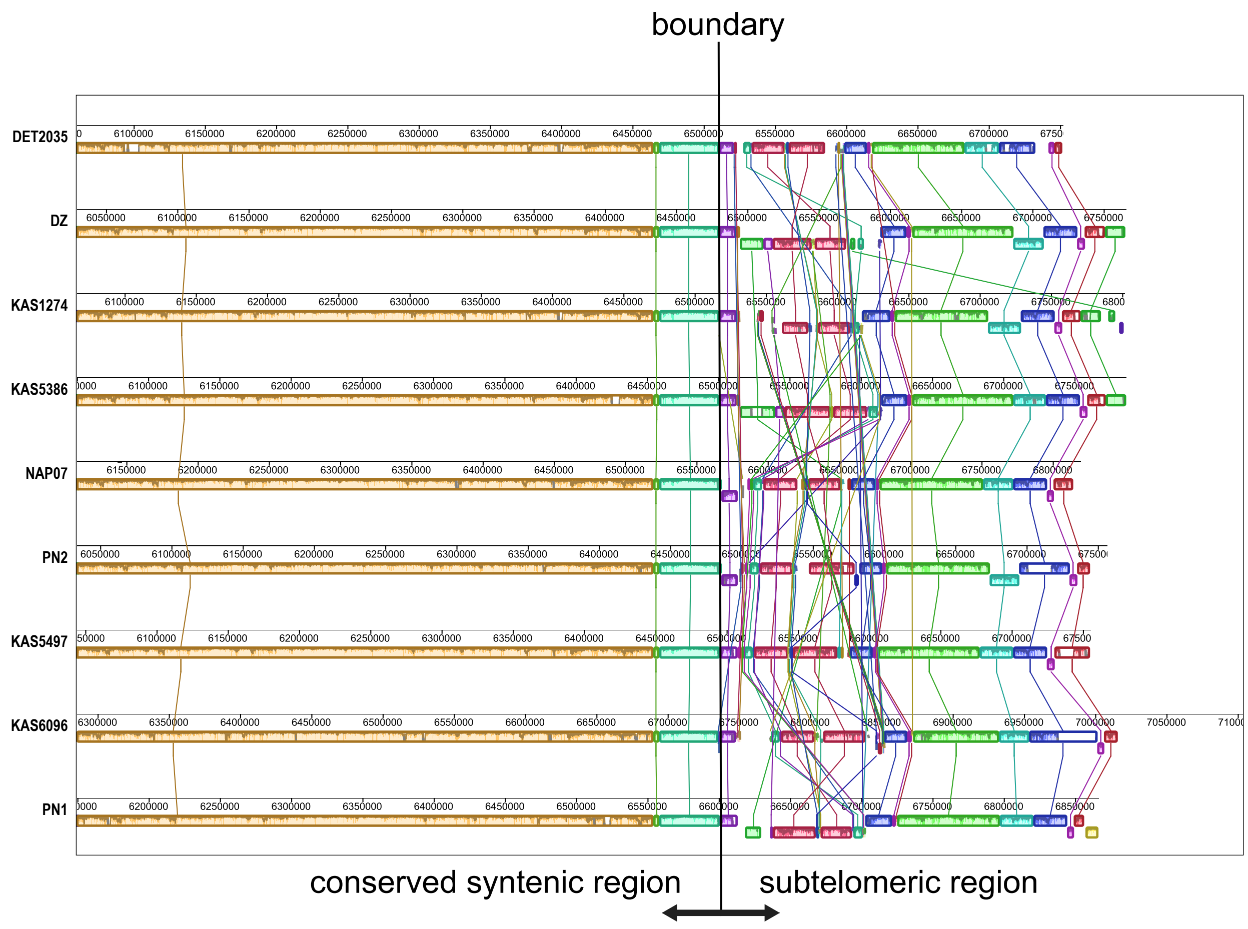

SUPPLEMENTARY FIGURE S2: Example of the demarcation of the subtelomere boundary using a chromosome alignment produced by progressiveMAUVE. The diagram shows the right terminus of Chromosome 01. The subtelomere boundary is placed at the alignment position where two or more genomes display moderately-sized insertions, deletions, and/or inversions.

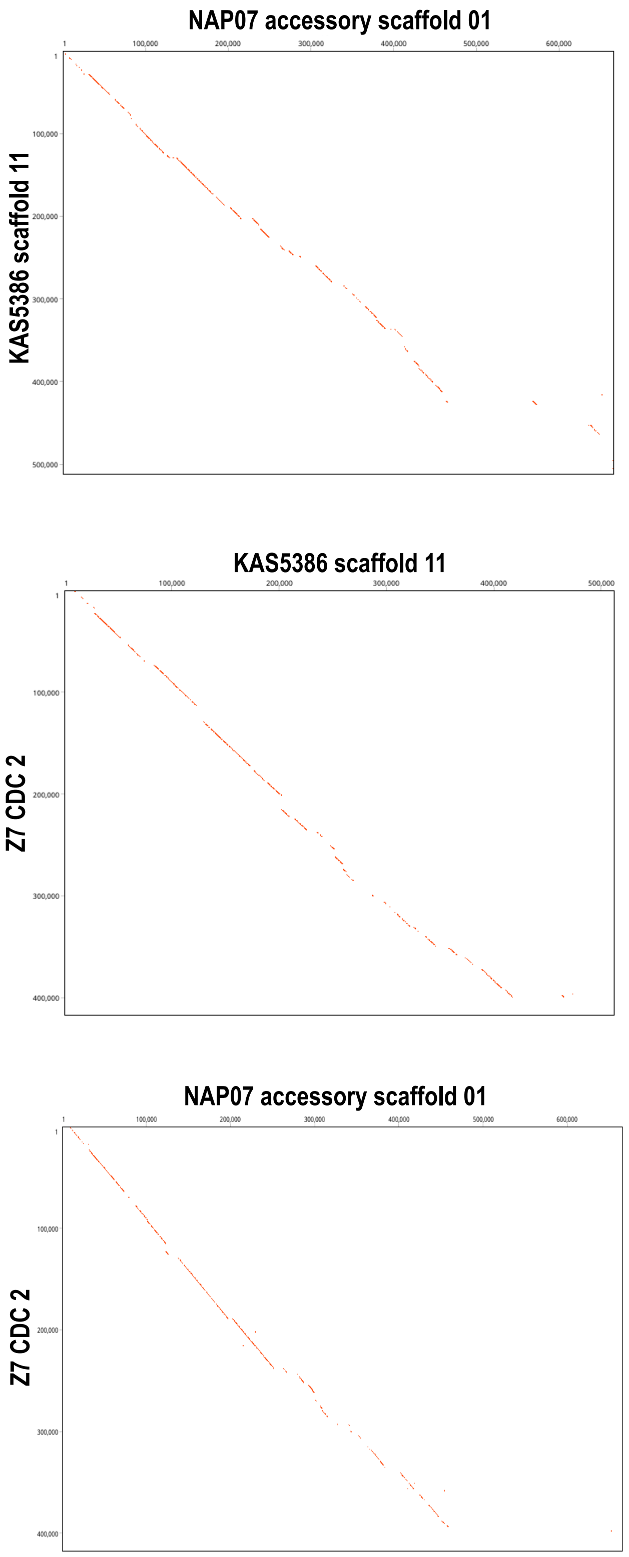

SUPPLEMENTARY FIGURE S3: Dotplots showing partial homology between putative accessory chromosomes from *A. alternata* KAS5386 (scaffold 11), *A. alternata* Z7 (CDC 2, CP061886.1), and *A. alternata* NAP07 (accessory scaffold 01, BJEP01000011.1). Plots were generated with Geneious Prime version 2025.0.3, EMBOSS dottup, word size of 100). Red dots indicate matches over 100 bp long.

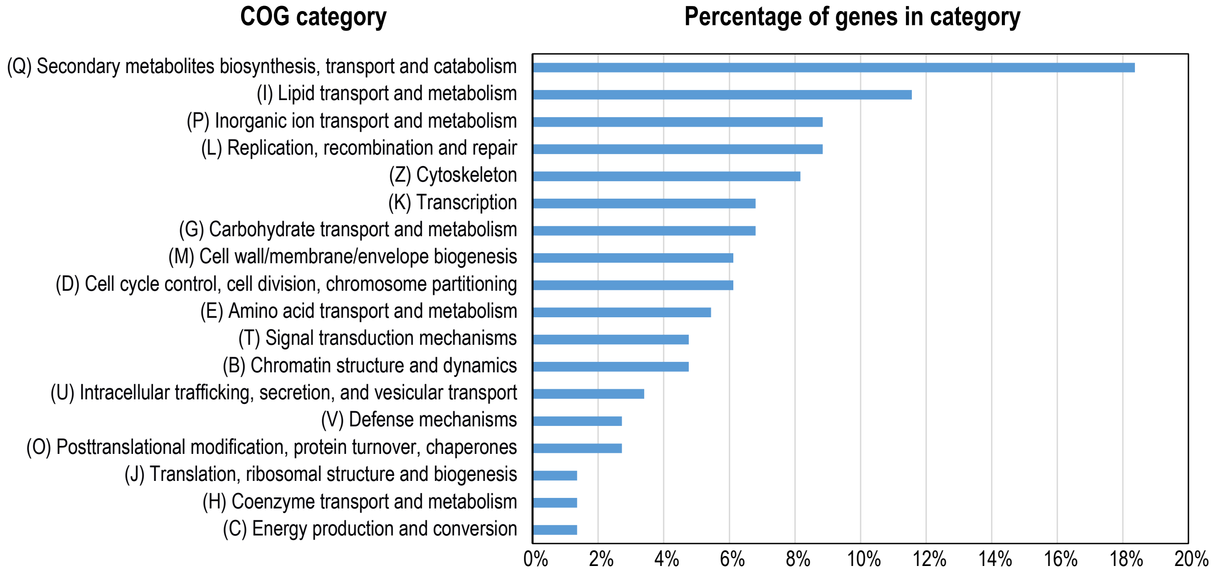

SUPPLEMENTARY FIGURE S4: Distribution of functional annotations for genes located in accessory genomic regions (five large-scale indels). Data are presented for the 147 (of 338) genes that were assigned to a COG (cluster of orthologous groups) category with a known function. Percentages add up to slightly more than 100% because somes genes were assigned to two COG categories.

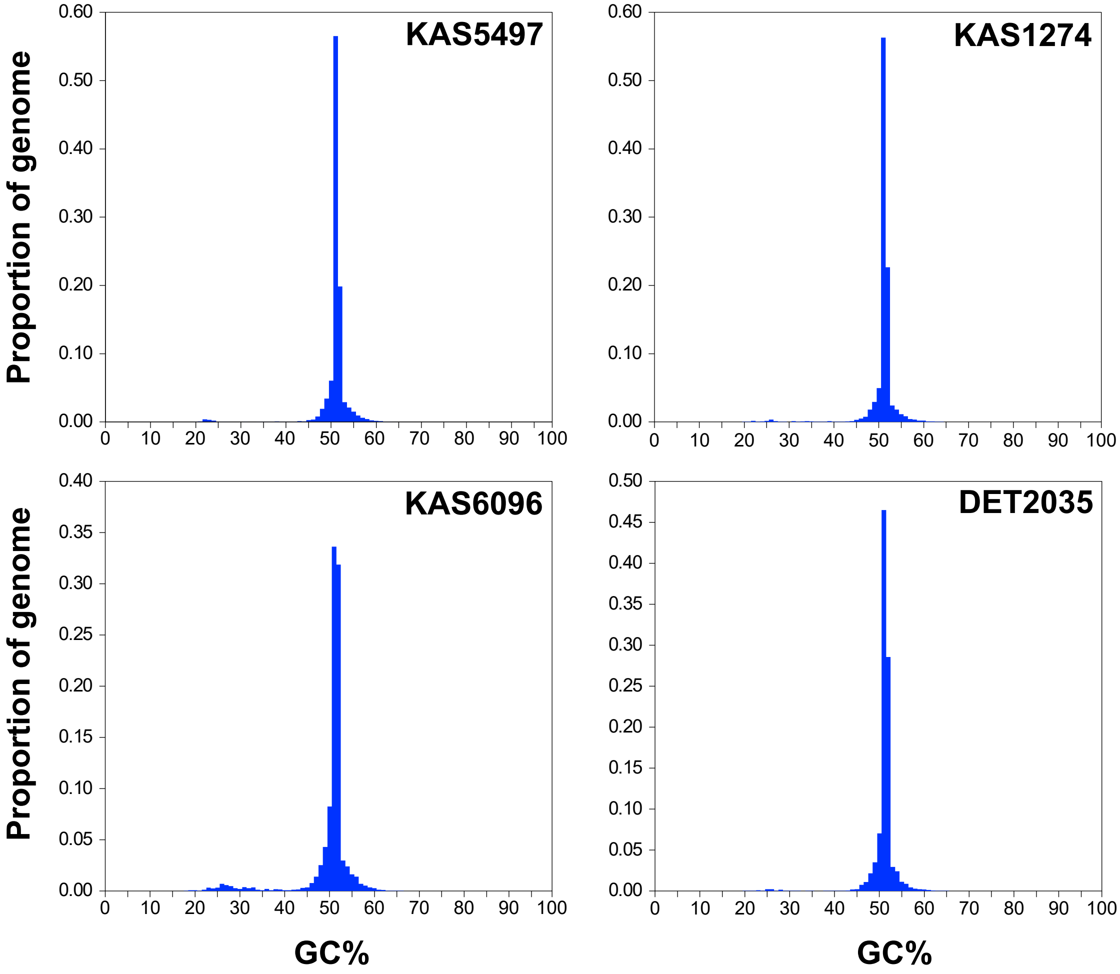

SUPPLEMENTARY FIGURE S5: Plots demonstrating a mostly uniform distribution of GC%, with very low amounts of AT-rich regions. Representattive genomes are shown for *A. alternata* KAS5497, *A. longipes* KAS1274, *A. arborescens* DET2035, and the undescribed sp. KAS6096. Even the genome with the highest proportion of AT-rich tracts (KAS6096) does not show evidence of distinct, bipartite compartmentalization.

SUPPLEMENTARY TABLE S1: Summary of sequence data outputs.

Strain	Number of Nanopore reads (mil)	Cumulative Nanopore output (Gb)	N50 of Nanopore reads (Kb)	Number of Illumina reads (mil)	Cumulative Illumina output (Gb)
DET2035	2.28	22.43	13.80	30.17	4.51
KAS1274	1.95	15.45	13.27	32.90	4.88
KAS5386	3.74	22.88	9.72	27.46	4.11
KAS5497	4.43	28.58	8.78	27.50	4.10
KAS6096	1.31	14.39	19.40	28.97	4.33
Average	2.74	20.75	12.99	29.40	4.39

SUPPLEMENTARY TABLE S2: Versions of NECAT assemblies chosen for subsequent polishing.

Three different combinations of PREP_OUTPUT_COVERAGE and CNS_OUTPUT_COVERAGE were tested for each genome: 50 and 40; 80 and 50; 100 and 60, respectively. The most contiguous of the three assembly versions was retained.

**Strain**	**GENOME_SIZE setting (Mb)**	**Chosen combination of PREP_OUTPUT_COVERAGE and CNS_OUTPUT_COVERAGE**
DET2035	33.5	100 and 60
KAS1274	34.1	80 and 50
KAS5386	34.4	80 and 50
KAS5497	34.4	80 and 50
KAS6096	35.7	50 and 40

SUPPLEMENTARY TABLE S3: Cross-validation of NECAT assemblies with CANU assemblies.

Different assembly algorithms may have particular strengths or weaknesses regarding assembly contiguity, so we cross-validated the outputs from NECAT and CANU assemblers. In general, the NECAT assemblies were more contiguous but did not conflict with the CANU assemblies. The most common difference was the failure of CANU to join the contigs for each arm of the chromosome, likely due to the repetitive, AT-rich nature of the centromeric regions.

Strain	Major structural differences found in CANU assemblies
**DET2035**	Chr03 as two contigs, Chr07 as two contigs.
**KAS1274**	Chr07 as two contigs.
**KAS5386**	Chr02 as two contigs, Chr05 as two contigs, Chr06 as two contigs, Chr07 as two contigs, Chr08 as two contigs.
**KAS5497**	Chr02 as three contigs, Ch03 missing right terminus (~142 kb), Chr04 as two contigs, Chr05 as three contigs, Chr08 as four contigs, Chr09 as three contigs.
**KAS6096**	Chr01 as three contigs, Chr02 as four contigs, Chr04 as three contigs, Chr05 as three contigs, Chr06 as two contigs, Chr07 as two contigs, Chr08 as five contigs, Chr09 as five contigs, Chr10 as three contigs.

SUPPLEMENTARY TABLE S4: Chromosomal homology relationships.

Using the established chromosomal nomenclature of *A. alternata* NAP07, chromosomes in existing genome assemblies were re-ordered, re-named, and reverse-complemented when appropriate. Note the numbering order of chromosomes does not strictly match a descending length pattern. In particular, Ch06-Ch09 have similar lengths so minor differences can lead to length order changes (e.g. in some genomes, Ch09 is longer than Ch08). The table below shows the correspondence between our unified chromosome names and those used in the original assemblies.

	**DZ**	**JS-0527**	**PN1**	**PN2**	**Y784-BC03**	**Z7**
**Unified chromosome name**	**Original name**	**Original name**	**Original name**	**Original name**	**Original name**	**Original name**
Ch01	chromosome 1 (revcomp)	Scaffold1	tig1	tig1	chromosome 1	chromosome 1
Ch02	chromosome 2 (revcomp)	Scaffold2	tig5	tig3	chromosome 2 (revcomp)	chromosome 2 (revcomp)
Ch03	chromosome 8	Scaffold3	tig18 (revcomp)	tig9	chromosome 3 (revcomp)	chromosome 3
Ch04	chromosome 5	Scaffold4 (revcomp)	tig20 (revcomp)	tig13	chromosome 4	chromosome 4 (revcomp)
Ch05	chromosome 4 (revcomp)	Scaffold5	tig6753	tig11	chromosome 5	chromosome 5
Ch06	chromosome 7	Scaffold6	tig33 (revcomp)	tig18 (revcomp)	chromosome 6	chromosome 6
Ch07	chromosome 6 (revcomp)	Scaffold7	tig26 (revcomp)	tig28	chromosome 7	chromosome 7 (revcomp)
Ch08	chromosome 9 (revcomp)	Scaffold9 (revcomp)	tig6755	tig23	chromosome 9 (revcomp)	chromosome 9 (revcomp)
Ch09	chromosome 3 (revcomp)	Scaffold8 (revcomp)	tig30 (revcomp)	tig20 (revcomp)	chromosome 8	chromosome 8 (revcomp)
Ch10	chromosome 10 (revcomp)	Scaffold10 (revcomp)	tig6758 (revcomp)	tig12832	chromosome 10 (revcomp)	chromosome 10 (revcomp)

SUPPLEMENTARY TABLE S5: Chromosome lengths and telomeric repeat presence.

Lengths (bp) with more than 10% deviation from the chromosomal mean are shaded in grey.

L and R indicate that telomeric repeats were found on the left and right ends of chromosomes, respectively.

		Chromosome									
**Strain**		**Ch01**	**Ch02**	**Ch03**	**Ch04**	**Ch05**	**Ch06**	**Ch07**	**Ch08**	**Ch09**	**Ch10**
**DET2035**	**Length**	6,751,719	5,464,541	3,270,632	3,094,696	2,918,825	2,557,077	2,497,999	2,346,372	2,406,338	1,863,886
	**Telo**	L	R		R	R	L+R	L		L+R	R
**DZ**	**Length**	6,765,325	5,498,111	2,518,499	3,031,091	3,148,290	2,577,428	2,824,270	2,467,937	3,425,204	1,852,273
	**Telo**	L+R	L+R	L+R	L+R	L+R	L+R	L+R	L+R	L	L+R
**JS-0527**	**Length**	7,020,625	5,441,345	3,332,389	3,058,847	2,799,051	2,638,833	2,548,068	2,392,276	2,424,841	1,841,203
	**Telo**	L+R	R	R	L+R	L+R	R	L	L	L	L+R
**KAS1274**	**Length**	6,801,499	5,509,638	3,298,134	3,095,008	2,857,058	2,637,459	2,616,291	2,530,753	2,532,733	1,874,223
	**Telo**	L+R	L+R	L+R	L+R	L+R	L+R	L+R	L+R	L+R	L+R
**KAS5386**	**Length**	6,785,880	5,551,739	3,304,563	3,158,438	2,867,613	2,579,988	2,510,552	2,462,517	2,408,698	1,855,471
	**Telo**	L+R	L+R	L+R	L+R	L+R	L+R	L	L+R	L+R	L+R
**KAS5497**	**Length**	6,754,991	5,521,615	3,314,789	3,045,658	2,807,936	2,538,134	2,529,850	3,014,522	2,594,036	1,829,559
	**Telo**	L+R	L+R	L+R	L+R	L+R	L	L	L	L+R	R
**KAS6096**	**Length**	7,103,584	6,056,242	3,381,425	3,491,677	2,996,249	2,853,692	2,639,173	2,677,005	2,686,520	1,970,893
	**Telo**	L+R	L+R	L+R	L+R	L+R	L+R	L+R	L	L+R	L+R
**NAP07**	**Length**	6,819,131	5,908,156	3,326,390	3,070,597	2,800,883	2,607,085	2,623,421	2,399,115	2,433,500	1,821,462
	**Telo**	L+R	L+R	L+R	L+R	L+R	L+R	L	L+R	L+R	L+R
**PN1**	**Length**	6,866,172	5,596,909	3,309,662	3,093,997	2,986,938	2,597,736	2,519,507	2,422,488	2,384,842	1,856,126
	**Telo**	L	L	L+R	L	L+R	L+R		L+R	L+R	L+R
**PN2**	**Length**	6,756,145	5,557,656	3,309,226	3,099,206	2,809,863	2,570,644	2,547,335	2,319,089	2,422,913	1,862,287
	**Telo**	L+R	L+R	L+R	L+R	L	L+R		R	L+R	L+R
**Y784-BC03**	**Length**	6,758,422	5,558,083	3,517,007	3,075,098	2,923,236	2,603,968	2,544,088	2,451,450	2,480,139	1,863,307
	**Telo**	L+R	L+R	L+R	L+R	L	L+R	L	L+R	L+R	L+R
**Z7**	**Length**	6,770,053	5,542,836	3,269,484	3,085,453	2,863,349	2,544,158	2,519,108	2,401,432	2,464,928	1,841,737
	**Telo**	L+R	L+R	L+R	L+R	L+R	L+R		R	L+R	L+R

SUPPLEMENTARY TABLE S6: Estimated lengths (kb) of subtelomeric regions.

	Ch01	Ch01	Ch02	Ch02	Ch03	Ch03	Ch04	Ch04	Ch05	Ch05	Ch06	Ch06	Ch07	Ch07	Ch08	Ch08	Ch09	Ch09	Ch10	Ch10
	Left	Right	Left	Right	Left	Right	Left	Right	Left	Right	Left	Right	Left	Right	Left	Right	Left	Right	Left	Right
DET2035	159	241	208	272	229	208	189	234	176	358	62	227	219	18	168	171	137	254	179	264
DZ	140	285	221	255	359	198	159	203	156	357	74	202	182	344	174	203	129	241	180	230
JS-0527	193	474	207	230	272	215	166	218	174	287	60	232	194	36	164	166	120	257	189	212
KAS1274	153	284	220	263	239	217	206	201	164	316	50	231	195	127	154	338	241	268	163	287
KAS5386	166	285	206	266	235	218	220	236	199	314	55	223	180	25	167	190	139	247	182	233
KAS5497	153	260	210	246	244	231	180	203	182	294	46	204	186	59	144	178	131	238	173	219
KAS6096	214	368	562	256	238	297	541	211	205	319	110	252	201	62	223	210	206	325	217	247
NAP07	150	251	215	275	225	213	179	209	168	298	58	256	189	134	182	186	143	271	170	220
PN1	130	265	206	237	243	201	178	218	179	454	63	233	159	76	187	191	135	243	178	235
PN2	156	270	245	243	237	202	182	217	171	276	67	200	182	50	134	158	142	233	170	240
Y784-BC03	176	247	209	276	241	218	200	210	202	329	71	200	190	49	167	210	135	248	199	221
Z7	180	251	205	291	233	210	202	212	169	322	55	210	106	109	149	181	130	227	175	217

SUPPLEMENTARY TABLE S7: Summary statistics for chromosomes, averaged across all twelve genomes.

Chromosome	Mean length	Genes per 50kb	BGCs per 1.0Mb	Transposable and repetitive elements per 50kb	Average IGOR
Ch01	6,829,462	17.75	1.11	8.57	0.925
Ch02	5,600,573	17.93	0.91	8.29	0.922
Ch03	3,262,683	17.61	0.87	9.09	0.931
Ch04	3,116,647	17.59	1.52	9.29	0.926
Ch05	2,898,274	17.19	0.35	11.06	0.926
Ch06	2,608,850	16.71	1.25	9.87	0.906
Ch07	2,576,639	17.24	1.16	10.41	0.933
Ch08	2,490,413	16.34	1.54	11.11	0.893
Ch09	2,555,391	17.09	1.92	10.80	0.908
Ch10	1,861,036	16.84	0.90	10.33	0.915

SUPPLEMENTARY TABLE S8: Numbers of secreted proteins and candidate effectors.

		All genes	Proteins with signal peptides	Candidate effectors without transmembrane domains
A. alternata	KAS5497	11839	1169	398
A. arborescens	DET2035	11567	1162	390
A. longipes	KAS1274	11732	1163	393
undescribed sp.	KAS6096	11866	1144	386

Supplementary Text:

SUPPLEMENTARY TEXT 1: Polishing assemblies

Polishing with Nanopore reads was performed with Racon (1.5.0) using minimap2 (2.27, Li 2018) mapping. Polishing with Illumina reads was performed with Pilon (1.23) using bwa-mem (0.7.17, Li 2013) mapping. Prior to polishing, short-read Illumina reads were trimmed with Trimmomatic v0.38 (Bolger et al 2014) under ILLUMINACLIP 2:30:10, TRAILING:20, and MINLEN:50.

SUPPLEMENTARY TEXT 2: Repeat annotation

Repeat types were annotated and masked (RepeatModeler, v2.0.1; RepeatMasker (v4.1.2.-p1) in priority of prediction robustness:

Round i) Low complexity/simple repeats were softmasked by RepeatMasker with the <-noint -xsmall> flag activated. Some annotation programs are designed to interpret and analyze softmasked regions differently as simple repeats may be incorporated in other repetitive or genomic elements downstream.

Round ii) Low complexity/simple repeats were skipped using the <-nolow> flag, as with all subsequent RepeatMasker rounds. Repeat consensus sequences for fungi from the curated RepBase library (20181026 Edition) were incorporated with the <-species fungi> flag.

Round iii) For strain-specific known elements, the *de novo* known repeat library created with RepeatModeler was provided with the <-lib> flag. Only complex and interspersed repeats were identified using the <-nolow> flag.

Round iv) For strain-specific unknown elements, the *de novo* unknown repeat library created with RepeatModeler was provided with the <-lib> flag. Only complex and interspersed repeats were identified using the <-nolow> flag.

SUPPLEMENTARY TEXT 3: Calculation of inter-genomic ortholog retention (IGOR) values

First, gene prediction must be performed, ensuring that the resulting gene names are unique and do not overlap between genomes (e.g. Genome001_Gene001 instead of just Gene001). Run OrthoFinder on protein files and locate the “Orthologues” output folder which contains a sub-folder for each genome. Within each sub-folder, there should be ortholog (.tsv) files that list the one-to-one, one-to-many, and many-to-many orthologs for each pairwise genome comparison with the focal genome (n-1). A focal gene will be present in an ortholog file only if it has an ortholog in the other genome, and will only occur once per ortholog file. For a focal gene ($FocalGene) in a focal genome ($FocalGenome), the number of other genomes that possess an ortholog (IGOR_Count) can be determined by a simple search of all ortholog files in the focal genome sub-folder:

```
IGOR_Count=$( grep $FocalGene
../Orthologues/Orthologues_$FocalGenome.proteins/*.tsv | wc -l )
```

This command can be incorporated into a standard loop or wrapper to calculate IGOR_Count for each focal gene in a genome. The IGOR_Value for the focal gene is

calculated by dividing the IGOR_Count by the number of pairwise comparisons (n-1), with results ranging from zero (no orthologs in any other genomes) to 1.0 (orthologs in all other genomes).

SUPPLEMENTARY TEXT 4: Manual joining of overlapping contigs for *A. alternata* KAS5386

The 10 core chromosomes and mitochondrial genomes could be identified as gapless contigs in all final NECAT assemblies, except for *A. alternata* KAS5386. For KAS5386, the assembly consisted of 15 non-mitochondrial contigs. Mapping of these contigs to each other revealed three instances where two contigs had significant lengths of identical sequence overlap at the contig ends (overlaps of 23.35 Kb, 20.69 Kb, and 16.24 Kb). Manual joining of these three contig pairs resulted in the full complement of 10 core chromosomes for KAS5386.

SUPPLEMENTARY TEXT 5: Further investigation of structural differences

For additional evidence supporting the translocations that were identified in, and unique to, the DZ assembly, we generated a new assembly from the existing DZ long-read sequence data (NCBI accession SRR24187893). Acknowledging that thresholds for contig-joining likely differ between assemblers, we used an assembler (NECAT) that was different than that used to create the original assembly (NextDenovo). If errors did exist, they were not likely to be identical between the two different assembly algorithms. Our NECAT assembly matched the original DZ assembly, producing similar contigs corresponding to the same full-length chromosomes. Comparison of assembly versions for the two chromosomes involved in the translocation (Ch03 and Ch09) revealed no structural differences between versions, supporting the original assembly. For Ch04 of the KAS6096 assembly and Ch08 of KAS5497 assembly, raw Nanopore reads were mapped (minimap2, 2.27) back to the full chromosome sequences. Coverage depth per site was calculated using the Samtools (1.9) <depth> command. No gaps in coverage were observed and coverage was not significantly lower in putative breakpoint regions.

SUPPLEMENTARY TEXT 6: Demarcation of subtelomeric regions

Homologous chromosomes from all twelve genomes were aligned using progressiveMAUVE (2.4; Darling et al. 2010) and the ends were visually inspected to determine the point at which synteny conservation begins to breakdown. Alignment regions with locally conserved synteny in all genomes were considered core, and the start of the subtelomeric region was the alignment position where two or more genomes displayed insertions, deletions, and/or inversions (see example in Supplementary Figure S2). Subtelomere length equals the distance from the subtelomere boundary to the end of each chromosome. Technically, the telomere repeats themselves occupy the terminus of each chromosome. These variable telomere lengths were not subtracted from the subtelomere length because they were very short (~130 bp) and therefore did not impact the kilobase-level estimates for subtelomere length. To confirm the position of the subtelomere boundary, analyses were repeated using homologous chromosome alignments generated by LASTZ (7.0.3; Harris 2007).

References:

Bolger AM, Lohse M, Usadel B. 2014. Trimmomatic: A flexible trimmer for Illumina sequence data. Bioinformatics, 30:2114–2120.

Darling, A. E., Mau, B., & Perna, N. T. (2010). progressiveMauve: Multiple genome alignment with gene gain, loss and rearrangement. PLoS ONE, 5:e11147.

Harris, R.S. (2007) Improved pairwise alignment of genomic DNA. Ph.D. Thesis, The Pennsylvania State University.

Li, H. (2013). Aligning sequence reads, clone sequences and assembly contigs with BWA-MEM. ArXiv:1303.3997v2. http://arxiv.org/abs/1303.3997

Li, H. (2018). Minimap2: Pairwise alignment for nucleotide sequences. Bioinformatics, 34:3094–3100.
